# Identification of WNK Gene in *Salvia miltiorrhiza* Reveals *SmWNK*7 Positively Regulates Root Growth and Salt Tolerance

**DOI:** 10.3390/plants15162438

**Published:** 2026-08-11

**Authors:** Yaqian Zhang, Yongxin Zhang, Zipeng Zhou, Wei Liu, Heng Lu, Xiao Wang, Mei Jiang

**Affiliations:** 1 Key Laboratory for Natural Active Pharmaceutical Constituents Research in Universities of Shandong Province, School of Pharmaceutical Sciences, Qilu University of Technology (Shandong Academy of Sciences), Jinan 250014, China; 2 Shandong Engineering Research Center for Innovation and Application of General Technology for Separation of Natural Products, Shandong Analysis and Test Center, Qilu University of Technology (Shandong Academy of Sciences), Jinan 250014, China

**Keywords:** *Salvia miltiorrhiza*, WNK gene family, root growth, salt stress, *SmWNK*7

## Abstract

The dried roots and rhizomes of *Salvia miltiorrhiza* are widely used and economically important traditional Chinese medicinal materials. Land salinization affects the growth of *S. miltiorrhiza*, resulting in a decline in its quality and yield. WNK kinases belong to a unique family of serine/threonine kinases. They act as key regulators of plant growth, development and abiotic stress responses. However, this gene family has not been systematically characterized in *Salvia miltiorrhiza*. In this study, nine *SmWNK* genes were identified at the whole-genome level in *S. miltiorrhiza*. Phylogenetic analysis classified them into four structurally conserved subgroups. These genes are distributed across eight chromosomes and contain two pairs of intraspecific syntenic genes. Interspecific collinearity is far stronger between *S. miltiorrhiza* and dicots than between *S. miltiorrhiza* and monocots. Cis-element prediction indicated these cis-elements participate in light signaling, hormone responses, stress responses and developmental regulation. Quantitative real-time PCR revealed that eight *SmWNK* genes were significantly induced by salt stress, and *SmWNK*7 was selected as the key candidate for functional validation. Functional assays via heterologous overexpression in tobacco demonstrated that *SmWNK*7 overexpression promoted root elongation and enhanced salt tolerance. Compared with wild-type tobacco plants, *SmWNK*7-overexpressing transgenic tobacco lines had higher catalase (CAT) and peroxidase (POD) activities, lower malondialdehyde (MDA) content, and stronger root viability. These changes alleviated oxidative damage by enhancing the antioxidant defense system. Yeast two-hybrid screening yielded 40 *SmWNK*7-interacting annotated proteins, including 6 transcription factors and 1 protein kinase, which were enriched in 81 GO terms and 27 KEGG pathways. These findings confirm *SmWNK*7 positively regulates root growth and salt tolerance, laying a theoretical foundation for exploring *SmWNK* genes’ role in plant stress adaptation.

## 1. Introduction

The With No Lysine (WNK) kinase encodes a unique serine/threonine protein kinase [1]. Within plant-related research, this gene was successfully cloned and isolated from the plant *Arabidopsis thaliana* in 2002 [2]. Typical protein kinases contain a highly conserved catalytic lysine residue in subdomain II, which is essential for ATP binding and kinase activity. In contrast, WNK proteins lack this conserved lysine residue, where this site is substituted by other amino acids; hence, they are named “with no lysine” kinases [3]. In animal WNKs, the canonical lysine site is mainly replaced by cysteine or asparagine. In plant WNKs, this residue is alternatively substituted by asparagine, serine, or glycine [4]. Despite this substitution, WNK kinases remain functionally active and have been shown to participate in the regulation of plant growth, hormone responses, and adaptation to both biotic and abiotic stress [5].

In plants, WNK kinases have been reported to influence growth and development processes in various species [6]. For instance, they regulate flowering time in *A. thaliana* through the modulation of circadian rhythms, vacuolar H^+^-ATPase, and the photoperiod pathway [7]. Upland cotton WNKs are also well characterized for their roles in circadian rhythm control [8]. In *Oryza sativa*, the *OsWNK*1 gene family exhibits distinct response patterns to various abiotic stresses, including low temperature, high temperature, salt stress, and drought [9]. Transcriptome databases from *Glycine max* indicate that *G. max* WNK genes display tissue-specific expression patterns and are induced by salt stress [10]. *SlWNK*11 enhances the scavenging of reactive oxygen species (ROS) and stabilizes cellular homeostasis, which ultimately strengthens the resistance of tomato plants to drought and salt stress [11]. In peach, WNK genes may participate in fruit development and ripening [12].

*S. miltiorrhiza* is a perennial herb of the genus *Salvia* in the Lamiaceae family, and its dried roots and rhizomes serve as the primary medicinal tissues. This plant mainly produces two types of bioactive components, namely hydrophilic phenolic acids and lipophilic tanshinones. Phenolic acid components mainly include rosmarinic acid and salvianolic acid B, which have various pharmacological effects such as anticoagulation, antioxidation, and antithrombosis [13,14,15]. The tanshinone fraction covers dihydrotanshinone, cryptotanshinone, tanshinone I and tanshinone IIA. These substances possess antitumor, anti-inflammatory and bacteriostatic bioactivities [13,16,17,18]. Nevertheless, field-grown *S. miltiorrhiza* frequently suffers from multiple abiotic hazards: continuous cropping obstacles, drought, salinity and heavy metal pollution. Such stress conditions greatly hinder sustained increases in crop yield and medicinal quality [19,20]. WNK kinases are among the most important gene families involved in the regulation of cellular functions [5]. Nevertheless, no studies have been reported on the members of the WNK family in *S. miltiorrhiza* and their physiological roles.

In this study, we identified and comprehensively analyzed nine members of the WNK family in *S. miltiorrhiza*. The analysis covered their physicochemical properties, phylogenetic relationships, chromosomal localization, gene structures, conserved motifs, as well as promoter cis-acting elements. Furthermore, the expression profiles of these *SmWNK*s in response to salt stress were evaluated via qRT-PCR. Functional characterization of *SmWNK*7 was performed by overexpressing this gene in tobacco, revealing its role in root growth and salt stress response. This study provides a comprehensive examination of the *SmWNK* gene family in *S. miltiorrhiza* and lays a foundation for further exploration of the roles of WNK genes in response to abiotic stress.

## 2. Results

### 2.1. Genome-Wide Identification and Comprehensive Analysis of the SmWNK Gene Family in Salvia miltiorrhiza

Through homology sequence alignment and domain validation with WNK genes from *A. thaliana* (TAIR10-thale cress) and *O. sativa* (V7.0-rice), a total of nine *SmWNK* family members were identified in the *S. miltiorrhiza* genome (Table S1). They were renamed sequentially as *SmWNK*1 to *SmWNK*9 according to their chromosomal locations. We analyzed the physicochemical properties of *SmWNK*s. These properties include protein length, molecular weight, theoretical isoelectric point (pI), instability index, aliphatic index, and grand average of hydropathicity (GRAVY). The results showed that the protein lengths encoded by *SmWNK* genes ranged from 295 amino acids (*SmWNK*5) to 723 amino acids (*SmWNK*6), with corresponding molecular weights ranging from 33,749.37 Da (*SmWNK*5) to 81,630 Da (*SmWNK*6). The theoretical pI values of the proteins ranged from 4.78 to 6.02, indicating that they are all acidic proteins. Their instability indices varied from 35.45 (*SmWNK*5) to 61.09 (*SmWNK*3). Apart from *SmWNK*2 and *SmWNK*5, all other proteins had instability indices higher than 40. This suggests poor stability inside cells. The GRAVY values ranged from −0.625 (*SmWNK*3) to −0.404 (*SmWNK*2), indicating that *SmWNK* proteins are hydrophilic (Table 1). Transmembrane domain prediction revealed that none of the *SmWNK* proteins contain transmembrane domains (Figure S1). The three-dimensional structure prediction of *SmWNK*s was performed, as shown in Figure S2. Subcellular localization prediction indicates that five *SmWNK* proteins are predicted to reside in the nucleus, two in chloroplasts, and two in the cytoplasm (Table 1).

### 2.2. Phylogenetic Analysis of SmWNKs

**Figure 1:**
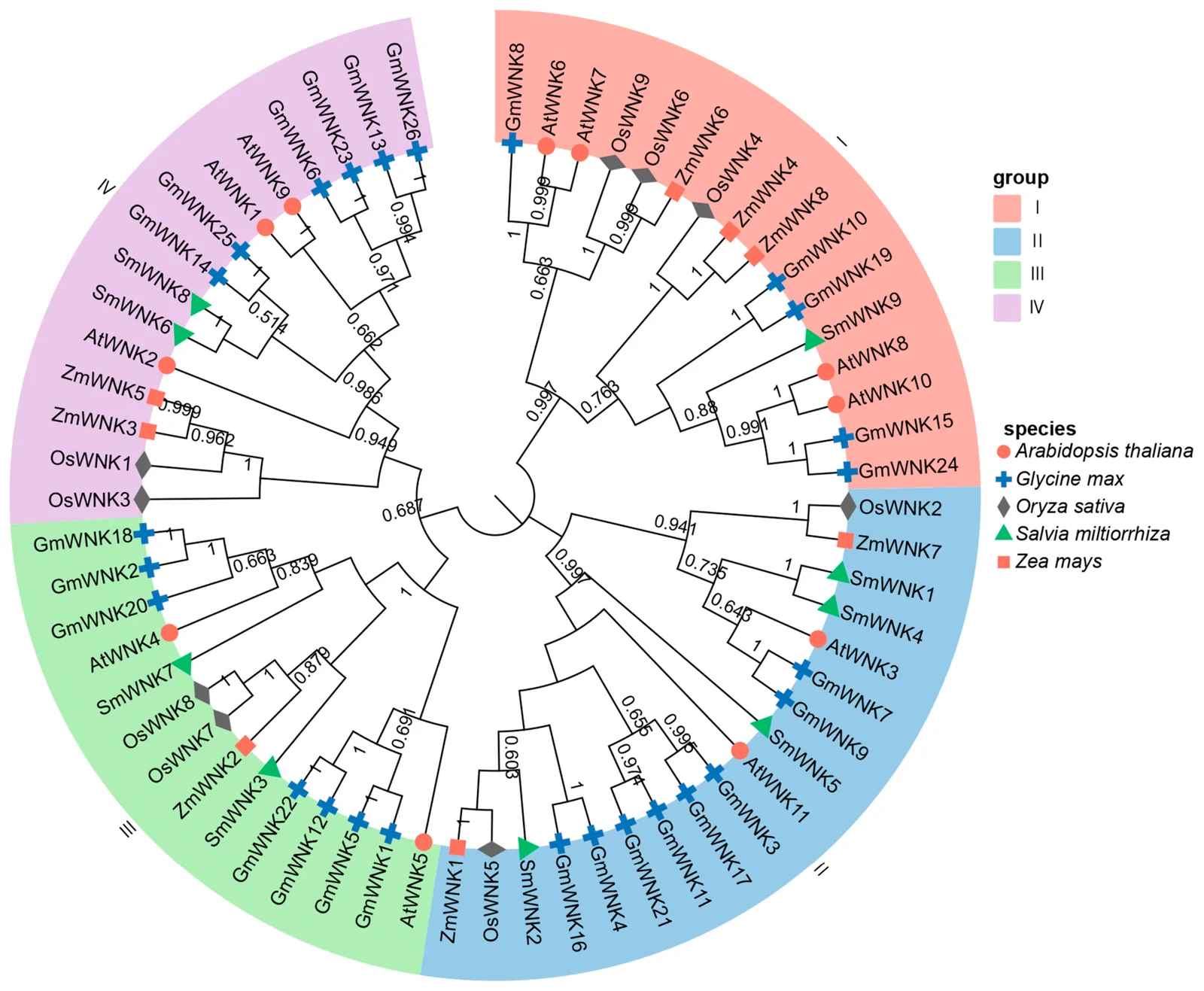
Maximum likelihood tree of phylogenetic relationships among plant WNKs. The WNK family was divided into four groups (I–IV), which were distinguished by different colors. Bootstrap values of 50% or higher supported by the labeled branch nodes are displayed. Different species are represented by different symbols. WNKs in *A. thaliana* are represented by orange circles, WNKs in *S. miltiorrhiza* by green triangles, WNKs in *G. max* by blue crosses, WNKs in *O. sativa* by gray diamonds, and WNKs in *Z. mays* by orange squares.

To explore the phylogenetic relationships among WNK family proteins, we constructed a phylogenetic tree. The tree included 63 WNK protein sequences from five species: *S. miltiorrhiza* (GWHDOEA00000000), *A. thaliana* (TAIR10-thale cress), *O. sativa* (V7.0-rice), *Zea mays* (RefGen V4), and *Glycine max* (Wm82.a2.v1) (Figure 1; Table S2). These WNKs were divided into four subgroups (Groups I–IV), containing 16, 18, 14, and 15 members, respectively. *SmWNK*9 belongs to Group I. *SmWNK*1, *SmWNK*4, *SmWNK*5 and *SmWNK*2 belong to Group II. *SmWNK*3 and *SmWNK*7 belong to Group III. *SmWNK*6 and *SmWNK*8 belong to Group IV. Pairwise *SmWNK* genes (*SmWNK*1/4 and *SmWNK*6/8) showed high internal node bootstrap values (100%) (Figure 1), suggesting that these genes may be paralogous and that functional redundancy may have arisen from gene duplication events.

### 2.3. Multiple Sequence Alignment of the SmWNK Proteins

Figure S3 presents the full-length amino acid sequence alignment of nine *SmWNK*s from *S. miltiorrhiza* and eleven *AtWNK*s from *A. thaliana*. All these WNK protein kinases exhibit high sequence conservation in the kinase catalytic domain region. The conserved motif “GXGXXK” contains the catalytic lysine (K) and corresponds to subdomain I. The sequence “GXEVAW” marks subdomain II. The motif “IIHRDLKCDNIFI” is observed in subdomain VIb, and the motif “GTPEFMAPE” is observed in subdomain VIII, confirming that these proteins belong to the Ser/Thr kinase group [21]. In addition to the kinase domain, the C-terminus contains an autoinhibitory conserved domain harboring an FXF motif [21].

### 2.4. Conserved Motifs and Gene Structure Analysis of SmWNKs

**Figure 2:**
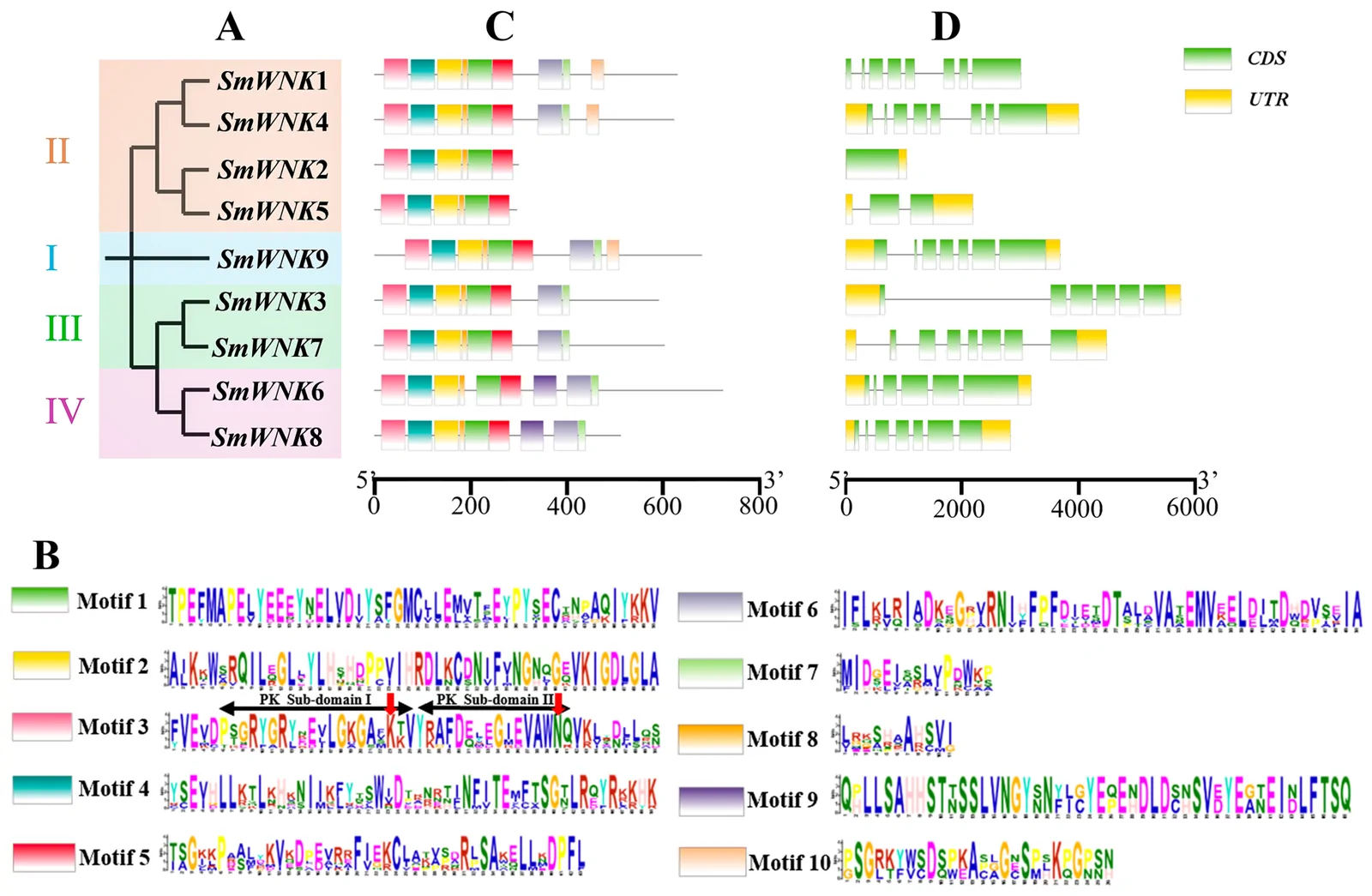
Analysis of phylogenetic tree, conserved motifs and gene structures of *SmWNK*s. (**A**) Phylogenetic tree of *SmWNK* proteins. (**B**) Sequences of 10 conserved motifs in *SmWNK*s. The height of each letter represents the relative frequency of the corresponding amino acid at that position. Based on previous research [22,23], subdomains I and II of protein kinases are denoted by the horizontal arrows above, and the positions of lysine (K) in subdomain I and the substituted lysine (K) in subdomain II are marked with arrows. (**C**) Conserved motif of *SmWNK* proteins. The different colored boxes represent different conservative motifs, with numbers from 1 to 10. The scale at the bottom indicates the protein length. (**D**) Structures of *SmWNK* genes. Exons are represented by green boxes and introns by black lines.

The nine *SmWNK* proteins were divided into four groups (Figure 2A). Ten conserved motifs were identified among the *SmWNK*s (Figure 2B,C). Most conserved motifs of *SmWNK*s are distributed in the N-terminal domain. They appear sequentially as motif 3, motif 4, motif 2, motif 8, motif 1, motif 5, motif 6, motif 7, motif 10, and motif 9. Six motifs, namely motif 1, motif 2, motif 3, motif 4, motif 5 and motif 8, are fully conserved across all *SmWNK* members. Notably, *SmWNK*3 and *SmWNK*7 (branch III) share identical motif compositions. The same pattern is also observed in *SmWNK*6 and *SmWNK*8 (branch IV). This further supports their close evolutionary conservation. Within motif 3, subdomain II lacks canonical lysine residue (K). This site is substituted by asparagine (N) or cysteine (C). By comparison, subdomain I contains the lysine residue. This feature matches the defining characteristic of “with no lysine” (WNK) kinases (Figure 2B).

The analysis of gene structures showed that *SmWNK*1 lacks UTRs, *SmWNK*2 possesses only a 3′UTR, and the remaining seven *SmWNK* genes contain both 5′UTR and 3′UTR. *SmWNK*2 and *SmWNK*5 have only 1–2 CDS segments, whereas the other *SmWNK* genes contain 6–8 CDS segments. Members of the same subfamily exhibited similarities in the numbers of exons and introns (Figure 2D).

### 2.5. Chromosomal Localization and Synteny Analysis of SmWNKs

**Figure 3:**
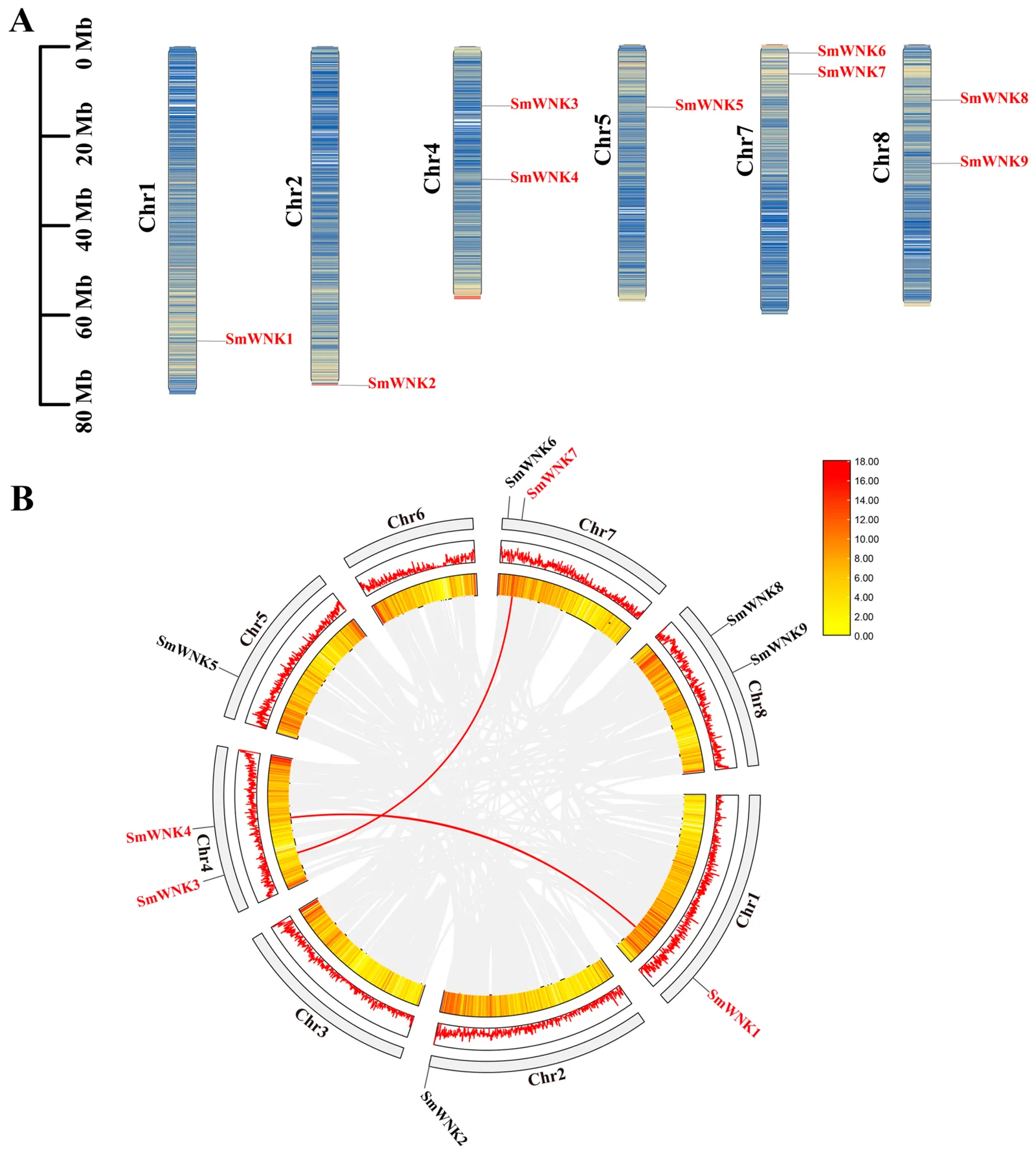
Chromosomal localization and intraspecific collinearity analysis of *SmWNK*s. (**A**) Chromosomal locations of the *SmWNK* gene family. Rectangles represent chromosomes. Chromosome names are shown in black on the left side of the rectangles, and gene names are shown in red on the right side of the rectangles. Chromosome lengths are displayed proportionally; (**B**) Collinearity analysis of the *SmWNK*s gene family. The fan-shaped rings indicate chromosome numbers and sizes. Gray line segments represent genomic collinear blocks, while red lines denote homologous gene pairs of *SmWNK* genes.

We extracted and analyzed the chromosomal locations of *SmWNK* genes and gene density on each chromosome of *S. miltiorrhiza* (Figure 3A). The results revealed that *SmWNK* family members are distributed on eight chromosomes. Chr1, Chr2, Chr4, Chr5, Chr7 and Chr8 each contain one or two *SmWNK* genes. No *SmWNK* genes were identified on the other two chromosomes.

**Figure 4:**
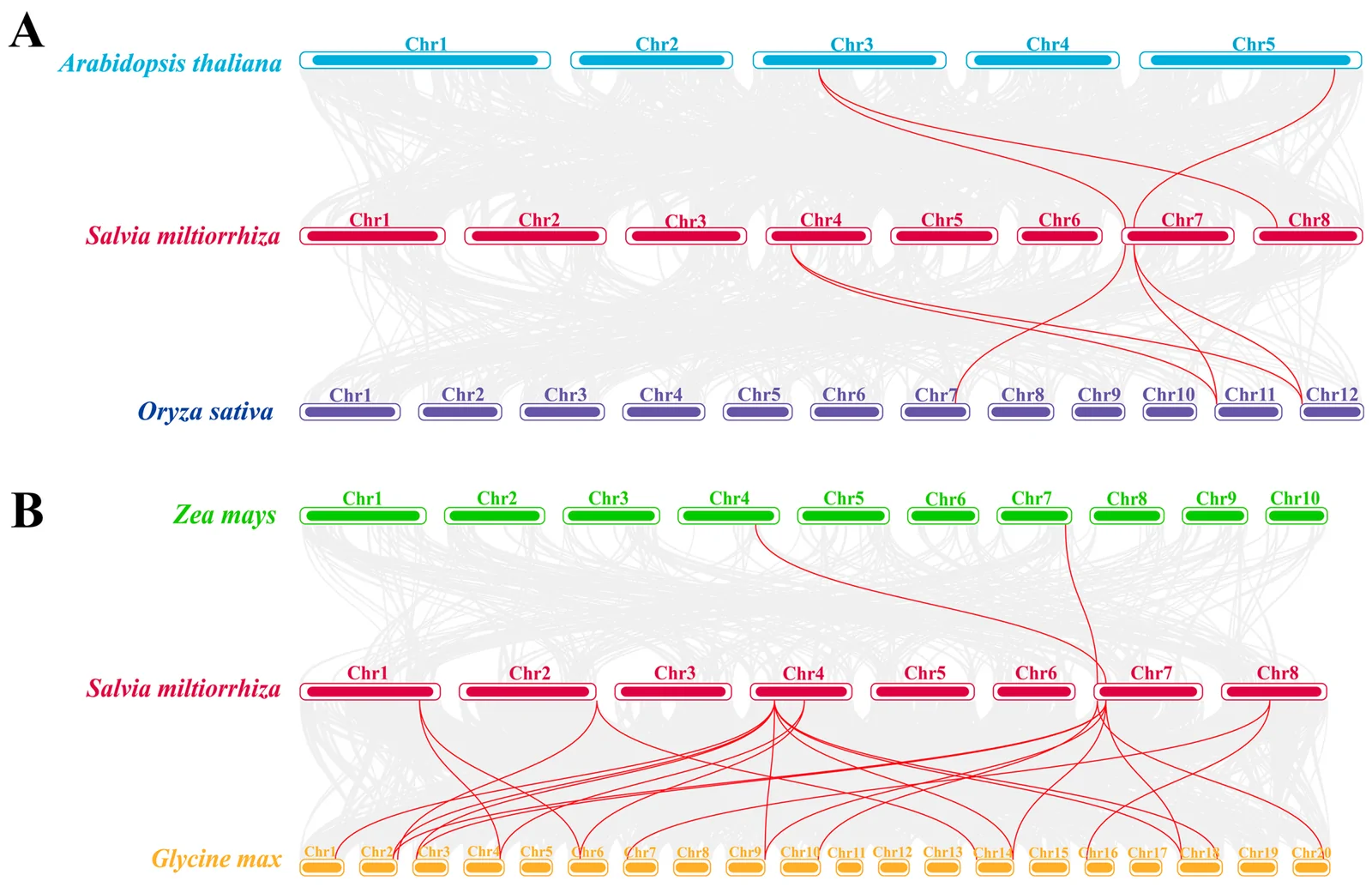
Collinearity analysis of the *SmWNK*s gene family in *S. miltiorrhiza* and 4 plant species. (**A**) Collinearity of *S. miltiorrhiza* with *A. thaliana* and *O. sativa*; (**B**) Collinearity of *S. miltiorrhiza* with *Z. mays* and *G. max*. Gray lines indicate the collinearity between the *S. miltiorrhiza* genome and those of *A. thaliana*, *O. sativa*, *Z. mays*, and *G. max*, while red lines highlight collinear gene pairs between *S. miltiorrhiza SmWNK* genes and genes from other species.

To elucidate the mechanism of *SmWNK* gene expansion in *S. miltiorrhiza*, intraspecific and interspecific synteny analyses were performed (Figure 3B; Figure 4). Among the nine *SmWNK* genes, two collinear gene pairs were identified, including *SmWNK*1 (Chr1) and *SmWNK*4 (Chr4), as well as *SmWNK*3 (Chr4) and *SmWNK*7 (Chr7). These results suggested that the expansion of the WNK gene family in *S. miltiorrhiza* can be attributed to segmental duplications. (Figure 3B) [24]. We further performed interspecific synteny analysis. The aim was to clarify evolutionary relationships between *SmWNK*s and their homologs from four other species: *A. thaliana*, *O. sativa*, *Z. mays*, and *G. max*. The results showed that *S. miltiorrhiza* shared 3, 5, 2, and 22 homologous gene pairs with *A. thaliana*, *O. sativa*, *Z. mays* and *G. max*, respectively, indicating that WNK homologous gene pairs may share a common ancestor and possess similar functions. The specific homologous gene pairs are listed in Table S3. In general, *SmWNK* genes display stronger syntenic relationships with dicots (*A. thaliana* and *G. max*). By contrast, synteny is weaker between *SmWNK*s and the monocots (*O. sativa* and *Z. mays*) (Figure 4).

### 2.6. Analysis of Cis-Acting Elements in the SmWNK Promoters

**Figure 5:**
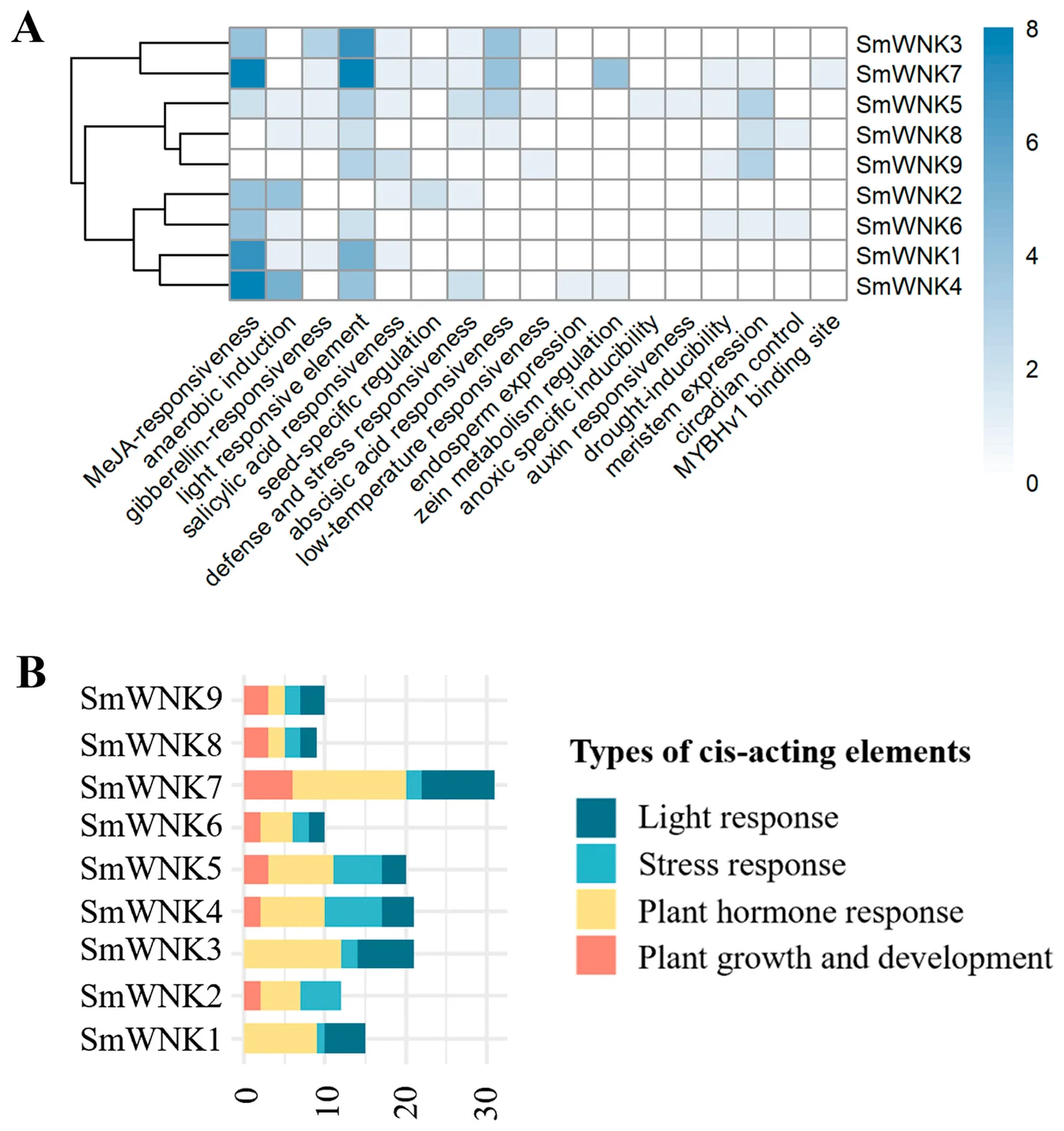
Promoter cis-acting elements of *SmWNK* genes. (**A**) The heatmap represents the distribution of the number of cis-acting elements; (**B**) The histogram shows the number of different types of cis-acting elements per gene, with different colors denoting distinct categories: light response, stress response, plant hormone response, and plant growth and development.

A total of 149 distinct types of cis-acting elements were predicted, belonging to 17 different categories (Figure 5A; Table S4). These elements were classified into four groups based on their association with light, hormones, stress, and development (Figure 5B). The number of elements in each group varied: 36 were related to light response, 65 to hormone response, 27 to stress response, and 21 to development (Tables S4 and S5).

Previous studies have demonstrated that certain WNKs are involved in plant hormone signaling pathways [8]. The promoters of *SmWNK*s contain five types of hormone-responsive elements: methyl jasmonate, gibberellin, salicylic acid, abscisic acid, and auxin, a composition similar to that of cis-acting elements in *G. max* [24]. Plant WNKs are also involved in stress tolerance [25]. In our predictions, *SmWNK* promoters additionally contain cis-acting elements associated with defense and stress-responsive, low-temperature-responsive, and drought-inducible elements. These findings suggest that *SmWNK*s may play important roles in plant development, hormonal stimulation, and stress tolerance.

### 2.7. Expression Profiles of the SmWNK Gene Family in Response to Salt Stress

**Figure 6:**
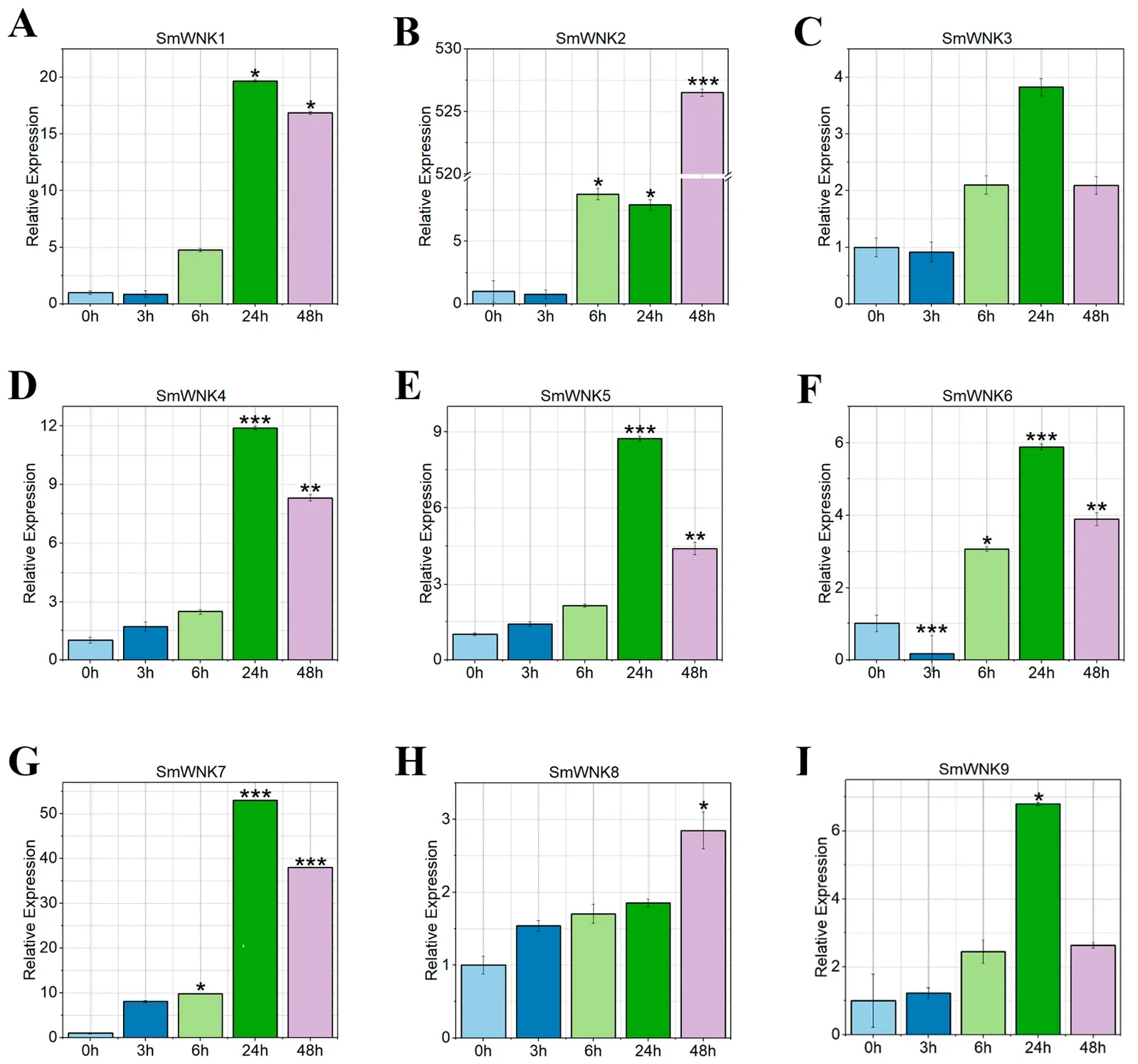
Relative expression levels of *SmWNK* genes after treatment with 200 mM NaCl. (**A**) Expression profile of *SmWNK*1; (**B**) Expression profile of *SmWNK*2; (**C**) Expression profile of *SmWNK*3; (**D**) Expression profile of *SmWNK*4; (**E**) Expression profile of *SmWNK*5; (**F**) Expression profile of *SmWNK*6; (**G**) Expression profile of *SmWNK*7; (**H**) Expression profile of *SmWNK*8; (**I**) Expression profile of *SmWNK*9. The *X*-axis represents the treatment time (0, 3, 6, 24, and 48 h), and the *Y*-axis represents the value of relative expression level. The 2^−ΔΔCT^ method was used for relative quantification. The values are expressed as the mean ± standard deviation (SD) of three replicates, where * indicates *p* < 0.05 vs. 0 h, ** indicates *p* < 0.01 vs. 0 h, and *** indicates *p* < 0.001 vs. 0 h.

To investigate the response of *SmWNK* genes to salt stress, qRT-PCR analysis was performed on the nine *SmWNK* genes (Figure 6). *S. miltiorrhiza* seedlings were treated with 200 mM NaCl, and samples were collected at 0, 3, 6, 24, and 48 h after treatment. The results showed that the induction levels of *SmWNK*s varied under salt stress. Compared with the 0 h time point, all genes except *SmWNK*3 were significantly upregulated after salt stress. Most genes (*SmWNK*1, *SmWNK*4, *SmWNK*5, *SmWNK*8, *SmWNK*9) showed significant upregulation at 24 h after salt stress, whereas a few genes (*SmWNK*2, *SmWNK*6, *SmWNK*7) exhibited significant upregulation as early as 6 h after salt stress. The expression levels of most genes (*SmWNK*1, *SmWNK*3, *SmWNK*4, *SmWNK*5, *SmWNK*6, *SmWNK*7, *SmWNK*9) peaked at 24 h after salt stress. These findings indicate that *SmWNK*s are involved in the response of *S. miltiorrhiza* to salt stress.

### 2.8. Effects of Overexpressing SmWNK7 on Seed Germination and Root Growth in Transgenic Plants Under Salt Stress

**Figure 7:**
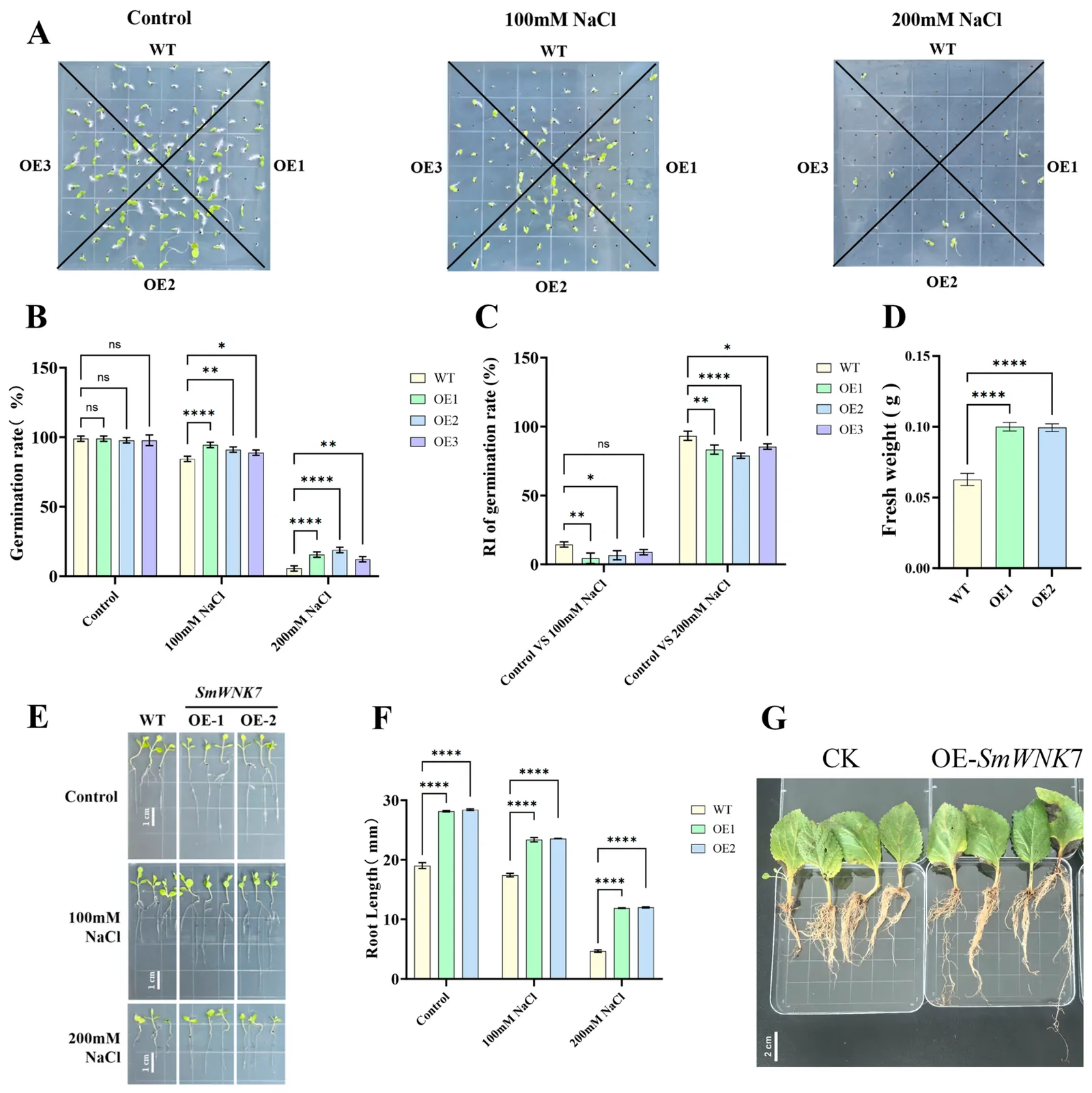
Effects of different salt stress on seed germination and root length of WT and OE-*SmWNK*7 tobacco lines. (**A**) Seed germination phenotypes under 0, 100, and 200 mM NaCl treatments in tobacco; (**B**) Statistical analysis of seed germination rate in tobacco; (**C**) Statistics of relative germination inhibition rate in tobacco; (**D**) Seedling fresh weight under 0 mM NaCl condition in tobacco; (**E**) Seedling root length phenotypes under 0, 100 and 200 mM NaCl treatments in tobacco; (**F**) Statistical analysis of root elongation under corresponding treatments in tobacco; (**G**) Phenotypic observation of root length in OE-*SmWNK*7 hairy roots of *S. miltiorrhiza*. Data are presented as mean ± SD from at least three biological replicates (*n* ≥ 3). Significant differences between OE lines and WT are indicated by asterisks (ns, no significant difference; * *p* < 0.05; ** *p* < 0.01; **** *p* < 0.0001).

qRT-PCR assays verified that salt stress significantly upregulated *SmWNK*7, and its relative expression level peaked at 50 at 24 h of salt treatment. Apart from *SmWNK*2, the remaining *SmWNK*s showed expression values lower than 20. We identified 33 cis-acting elements in the *SmWNK*7 promoter region. In comparison, promoters of the other *SmWNK*s carried merely 9–21 cis-acting elements (Table S5). Taken together, we designated *SmWNK*7 as the core candidate gene and dissected its function via heterologous overexpression in tobacco (Figure S4). Under normal conditions (0 mM NaCl), the seed germination rates of overexpression (OE) lines were comparable to those of the WT (Figure 7A–C). Nevertheless, OE lines displayed better overall growth than WT. Specifically, primary root elongation and fresh weight were significantly increased in OE lines relative to WT (Figure 7D–F). In the experiment of inducing hairy root formation via Agrobacterium infection at the base of single leaves of *S. miltiorrhiza*, four independent positive OE-*SmWNK*7 hairy root lines were generated, with empty vector pIB360 lines as the control (CK). Full root length measurements for all lines are provided in Supplementary Table S6. Phenotypic observations revealed that hairy roots of OE-*SmWNK*7 lines were markedly longer than those of CK (Figure 7G and Figure S5). These results indicate that *SmWNK*7 positively regulates plant vegetative growth and root development.

After salt stress treatment, seed germination rates and seedling growth were inhibited to varying degrees in all lines, but the OE lines exhibited a more pronounced salt-tolerant advantage. Under 100 mM and 200 mM NaCl stress, OE lines showed significantly higher seed germination rates and significantly lower relative germination inhibition rates compared with the WT (Figure 7B,C), along with a superior overall growth status (Figure 7A). Meanwhile, primary root elongation of the OE lines was significantly higher than that of the WT under both 0, 100 and 200 mM NaCl conditions (Figure 7E,F). The relative inhibition rate of root length was significantly lower in OE lines (57.68–57.76%) than in WT (75.36%) under 200 mM NaCl stress. These results demonstrate that *SmWNK*7 promotes root growth under normal conditions and effectively relieves the salt-induced inhibition of root growth under high-salt stress. This gene thereby maintains the structural and functional integrity of roots under salt stress.

### 2.9. Detection of Physiological Indices in OE-SmWNK7 Plants

**Figure 8:**
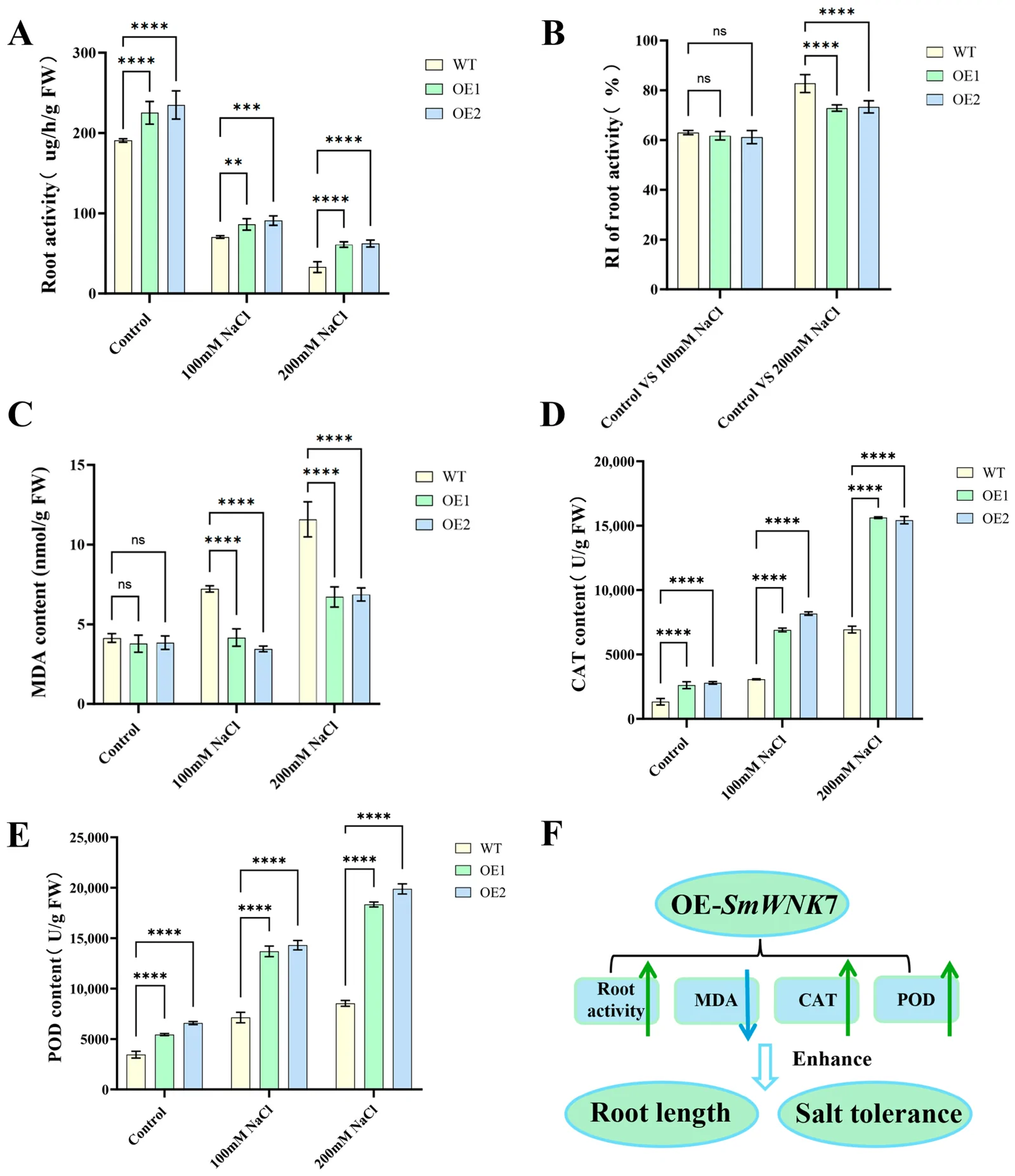
Physiological characterization of OE-*SmWNK*7 tobacco plants under salt stress. (**A**) Root activity of WT and OE-*SmWNK*7 lines under different NaCl concentrations (0, 100, 200 mM); (**B**) Relative inhibition rate of root activity; (**C**) Malondialdehyde (MDA) content; (**D**) Catalase (CAT) activity; (**E**) Peroxidase (POD) activity; (**F**) Functional schematic of OE-*SmWNK*7. Data are presented as mean ± SD from at least three independent biological replicates (*n* ≥ 3). Asterisks denote significant differences between overexpression lines and WT under the same treatment (** *p* < 0.01; *** *p* < 0.001; **** *p* < 0.0001; ns, not significant).

To further explore how *SmWNK*7 promotes growth and enhances salt tolerance, this study evaluated root activity, oxidative damage levels and antioxidant enzyme activities in tobacco. Root activity assays showed that OE lines exhibited significantly higher root activity than the WT under both normal (0 mM NaCl) and salt stress conditions (100 and 200 mM NaCl) (Figure 8A). Specifically, under 200 mM NaCl stress, the relative inhibition rate of root activity in OE lines was significantly lower than that in the WT, which was consistent with the reduced relative inhibition of root length at the same concentration (Figure 8B).

Malondialdehyde (MDA) content assay showed no significant difference in MDA content between WT and OE lines under normal conditions. After salt stress, MDA content increased in all lines, but OE lines accumulated significantly lower MDA levels (Figure 8C), indicating that *SmWNK*7 alleviates membrane lipid peroxidation damage. Antioxidant enzyme activity detection showed that the activities of Catalase (CAT) and peroxidase (POD) in OE lines were significantly higher than those in WT under both normal and salt stress conditions and remained superior with increasing salt concentration (Figure 8D,E), indicating stronger ROS scavenging and damage resistance abilities. In summary, *SmWNK*7 plays a dual role in promoting root development and conferring salt tolerance. Under normal conditions, it enhances root growth and root vitality. Under salt stress, *SmWNK*7 overexpression significantly increases CAT and POD activities and reduces MDA accumulation. These changes sustain high root vitality and mitigate salt-induced root growth inhibition, thereby positively regulating plant salt tolerance (Figure 8F).

### 2.10. Yeast Two-Hybrid Assay

To screen the interacting target proteins of *SmWNK*7, a yeast two-hybrid assay was performed using the GAL4 system in this study. The full-length sequence of *SmWNK*7 was fused to the GAL4 DNA-binding domain (BD) of the pGBKT7 vector. The recombinant vector BD-*SmWNK*7 was successfully transformed into yeast competent cells (Table S7; Figure S6). Self-activation verification confirmed that the optimal screening concentration for yeast hybridization was 500 ng/mL (Figure S7A). Subsequently, BD-*SmWNK*7 was hybridized with the *S. miltiorrhiza* cDNA yeast library. Blue positive colonies were picked and further verified by bacterial liquid PCR (Table S8; Figure S8) and Sanger sequencing. A total of 48 candidate target proteins were initially identified. Among them, SMil_00011043 (gene 7) occurred five times, SMil_00012167 occurred three times, and SMil_00027216 (gene 5), SMil_00005121 (gene 16) as well as SMil_00006036 (gene 21) each appeared twice. After redundancy removal combined with GO and KEGG functional enrichment analysis, 40 proteins with clear functional annotations were finally obtained (Supplementary File S1). Among these annotated proteins, six were transcription factors, specifically gene 2, gene 9, gene 23, gene 28, gene 30, and gene 35, while one was a protein kinase (gene 39). Furthermore, these functionally annotated proteins were significantly enriched in 81 GO terms and 27 KEGG pathways. Only core functional terms relevant to salt stress and root development are visualized in Figure S7B,C for better readability, and all complete enrichment datasets are provided in Tables S9 and S10.

## 3. Discussion

WNK proteins belong to the family of soluble serine/threonine kinases and are named after the presence of an essential catalytic lysine at an atypical position [26]. Compared with animals, plants possess a larger number of WNK genes [7], with significant variation among species. In this study, nine *SmWNK* genes were identified in *S. miltiorrhiza* through genome-wide analysis. All WNK gene sequences were confirmed based on knowledge of their atypical position of the lysine (K) residue (Figure 2). The N-terminal subdomain I of the kinase domain contains the consensus motif Gly-X-Gly-X-X-Gly-X-Val, whereas in WNK kinases the third glycine is replaced by a lysine (K) residue, changing the consensus motif to Gly-X-Gly-X-X-Lys-X-Val (Figure S6). This forms the single active center of *SmWNK*s, consistent with previous reports [9].

Gene structure prediction and subcellular localization prediction of the nine WNK family members in *S. miltiorrhiza* revealed that the amino acid numbers of the *SmWNK* gene family (ranging from 299 to 723 AA) (Table 1) are similar to those in *A. thaliana*, *O. sativa* and *G. max* [7,9,24]. In contrast, the human WNK1 protein consists of approximately 2382 amino acids, which is three times the length of *SmWNK*s [4]. Structural analysis showed *SmWNK*s contain 1 to 8 exons (Figure 2D). This pattern matches *G. max* WNK genes, which carry 2–8 exons [24]. Subcellular localization prediction analysis revealed that most *SmWNK* proteins were targeted to the nucleus, while a small number were localized to chloroplasts and cytoplasm (Table 1). This result is consistent with predictions for *O. sativa* using the mPLoc database. However, predictions for *O. sativa* using the mPLK database showed that all *OsWNK* proteins are localized in the cytoplasm, while predictions for *G. max GmWNK* proteins indicated distribution across different subcellular compartments [9].

Gene duplication is not only a major source of evolutionary innovation but also a primary driver of gene family expansion [27]. The phylogenetic tree (Figure 1) divided the WNK family into four clades. We compared *SmWNK*s with their homologous genes from other plant species to predict the potential functions of each *SmWNK* protein. These results lay a theoretical foundation for follow-up functional verification and mechanism research. In Group I, *AtWNK*6 regulates circadian rhythms [2], *AtWNK*8 controls flowering time and enhances tolerance to salt and osmotic stress [28,29], and *OsWNK*9 modulates responses to salt and drought stress through an ABA-dependent pathway [21]. Given the functional conservation of homologous WNK proteins, *SmWNK*9 within this clade likely shares these stress-related functions. In Group II, *O. sativa OsWNK*2 responds to drought and high-temperature stress, while *OsWNK*5 specifically responds to salt stress [9]. *SmWNK*1, *SmWNK*2 and *SmWNK*4 fall into this clade, so they are presumed to participate in abiotic stress adaptation in *S. miltiorrhiza*. In Group III, *GmWNK*1 confers strong salt and osmotic stress tolerance [10]. Thus, *SmWNK*3 and *SmWNK*7 from this clade may carry out identical roles, which need further experimental verification. In Group IV, *AtWNK*1 regulates circadian rhythms and flowering time [2], and *AtWNK*9 enhances drought tolerance by positively regulating ABA signaling [30]. Accordingly, *SmWNK*6 and *SmWNK*8 clustered here may perform comparable biological roles. All these functional predictions offer clear research directions. They support future work to dissect the exact molecular mechanisms through which core *SmWNK* proteins regulate stress response, plant growth and development in *S. miltiorrhiza*.

Synteny analysis revealed that *S. miltiorrhiza* shares 3, 5, 2, and 22 homologous gene pairs with *A. thaliana*, *O. sativa*, maize, and *G. max*, respectively (Figure 4; Table S3). Published studies show *AtWNK*2 and *AtWNK*4 separately control flowering time and circadian rhythms [2,28]. Thus, their orthologs *SmWNK*6, *SmWNK*8 and *SmWNK*7 likely retain these conserved biological roles. In addition, rice *OsWNK*1 participates in circadian clock control and abiotic stress adaptation simultaneously [31]. Its ortholog *SmWNK*6 is therefore presumed to carry this dual regulatory function. All these collinearity results demonstrate tight evolutionary connections across different plant species. Meanwhile, they also emphasize that gene duplication and collinear blocks drive WNK family expansion and maintain functional conservation during evolution.

Cis-acting elements regulate plant responses to hormones and various stresses during growth and development [32]. We scanned the promoter sequences of all *SmWNK* genes and identified abundant cis-regulatory elements. These motifs are associated with light responses, phytohormone signaling, plant growth and stress adaptation (Figure 5B). Published research confirms jasmonic acid (JA) and abscisic acid (ABA) govern multiple plant physiological pathways and boost stress resistance [25,33,34]. Both hormones are critical for coping with harsh environments including drought, salt and heat stress. The high frequency of abscisic acid and jasmonic acid-responsive elements in the promoters of *SmWNK* genes suggests an association between the WNK family and plant stress responses (Figure 5A).

We used qRT-PCR to detect *SmWNK* expression profiles under 200 mM NaCl salt stress (Figure 6). Within the 48-h treatment period, every *SmWNK* gene except *SmWNK*3 displayed markedly elevated transcript abundance relative to the 0 h control. Notably, *SmWNK*6 presented a dynamic expression trend. Its transcript level dropped sharply at 3 h, rose obviously at 6 h, and hit the maximum at 24 h. This kind of transient early repression has also been documented in other species. For instance, *GmWNK*1 [10] in *G. max* were suppressed at 2 h under salt stress. The rapid early-stage salt-stress response in plants is dominated by post-translational regulation such as protein-kinase phosphorylation rather than messenger-RNA accumulation [35]. qRT-PCR data have demonstrated that the moso-bamboo WNK homologue *PeWNK*9 is up-regulated 117-fold after 24 h NaCl-treatment. Accordingly, several plant WNK genes require sustained salt-signal input for maximal transcriptional activation [36]. The subsequent decline in transcript levels is governed by endogenous negative-feedback circuits, which make a trade-off between salt-defence activation and regular vegetative growth [37]. In contrast, the majority of genes in this study showed significant upregulation upon salt stress. Since *SmWNK*7 maintained relatively high transcript abundance under salt stress, we constructed its overexpression vector OE-*SmWNK*7 for further functional identification.

Seed germination and root length assays demonstrated that overexpression of the *SmWNK*7 gene promotes plant growth and enhances salt tolerance (Figure 7). Salt stress primarily inhibits seed germination, suppresses root growth, and impairs normal plant growth and development by inducing the accumulation of ROS [38]. Among the antioxidant enzymes, CAT and POD are key components [39,40]. Thiobarbituric acid reactive substances (TBARS) act as classic biomarkers of membrane lipid peroxidation, and MDA is their major constituent. MDA binds to thiobarbituric acid and generates chromogenic products. Accordingly, MDA quantification can mirror TBARS abundance and assess the degree of membrane oxidative injury. Lower MDA concentrations suggest milder oxidative injury and stronger salt resilience. Under control conditions, MDA concentrations showed no significant difference between WT and OE-*SmWNK*7 tobacco lines (OE1, OE2). Following NaCl exposure, MDA concentrations increased markedly in all tested plant lines. However, the overexpression lines retained substantially lower MDA concentrations than WT under both 100 mM and 200 mM NaCl treatments (Figure 8C). These results reveal that *SmWNK*7 overexpression relieves salt-induced membrane lipid peroxidation as evidenced by the suppression of MDA/TBARS buildup. Root activity directly determines root growth status and the plant’s ability to adapt to stress [41]. Subsequent physiological assays provide supporting evidence. Overexpressing *SmWNK*7 elevates CAT and POD activity, alleviates lipid peroxidation injury, relieves salt-triggered root growth suppression, and preserves normal root vigor. This physiological regulation supports seed germination and root growth, ultimately leading to coordinated positive regulation of tobacco growth and salt tolerance. These findings lay a foundation for stress-tolerance breeding in dicotyledonous plants, although further validation is needed for practical applications.

In this study, yeast two-hybrid screening of a cDNA library identified 40 candidate interacting proteins. Six of them are transcription factors from the bZIP (gene9, gene28), NAC (gene23, gene30) and BBX (gene2, gene35) families, and another one is a serine/threonine receptor-like cytoplasmic kinase of the RLCK-VII subfamily (gene39). Previous studies have confirmed that transcription factors of the bZIP, NAC and BBX families all positively regulate plant salt stress responses and root growth. In *A. thaliana*, plants overexpressing *AtbZIP*1 or *AtbZIP*17 show greatly improved salt tolerance, and salt-induced root growth inhibition is alleviated [42,43]. Wheat *TabZIP*15 regulates the glycolysis pathway and scavenges ROS to promote root growth under salt stress [44]. Tobacco *NtbZIP*62 responds to salt and ABA signals, and its overexpression can effectively restore primary root elongation under saline conditions [45]. NAC transcription factors can independently regulate ABA and auxin signals and simultaneously coordinate plant salt tolerance and root development [46]. Soybean *GmNAC*06 independently activates genes related to osmoprotectant synthesis and ion transport to maintain Na^+^/K^+^ homeostasis. Its overexpression obviously relieves salt damage to hairy roots [47]. *Tamarix hispida ThNAC*4 and *ThNAC*7 separately activate antioxidant and proline synthesis pathways. They eliminate excess ROS, protect root meristems and greatly increase root length under salt stress [48,49]. Overexpression of *AtBBX*24 in *A. thaliana* significantly rescues salt-suppressed root growth [50]. Overexpression of *IbBBX*24 in sweet potato markedly enhances plant salt tolerance [51]. RLCKs act as core hubs of plant stress signal transduction. They receive signals from upstream kinases and directly phosphorylate MAPKKKs to trigger the MAPK cascade [52,53]. Previous studies have verified the physical interaction between rice *OsWNK*1 and the receptor-like cytoplasmic kinase *OsRLCK*109, whereas their protein-protein complex exclusively modulates ROS homeostasis within leaf tissues [54]. Accordingly, we hypothesize that *SmWNK*7 mediates root salt stress responses by interacting with these candidate proteins via two pathways: transcriptional regulation and protein kinase signaling. Nevertheless, this study has clear limitations. Yeast two-hybrid screening only serves as an in vitro preliminary test. It may yield false positives and cannot reproduce genuine protein interactions inside native plant cells. Follow-up work will require multiple in vitro and in vivo tests for further validation, including pairwise yeast two-hybrid assays, bimolecular fluorescence complementation (BiFC), co-immunoprecipitation (Co-IP), pull-down assays, genetic complementation and root phenotyping. These experiments aim to verify the predicted protein interactions and the proposed regulatory hypothesis.

**Figure 9:**
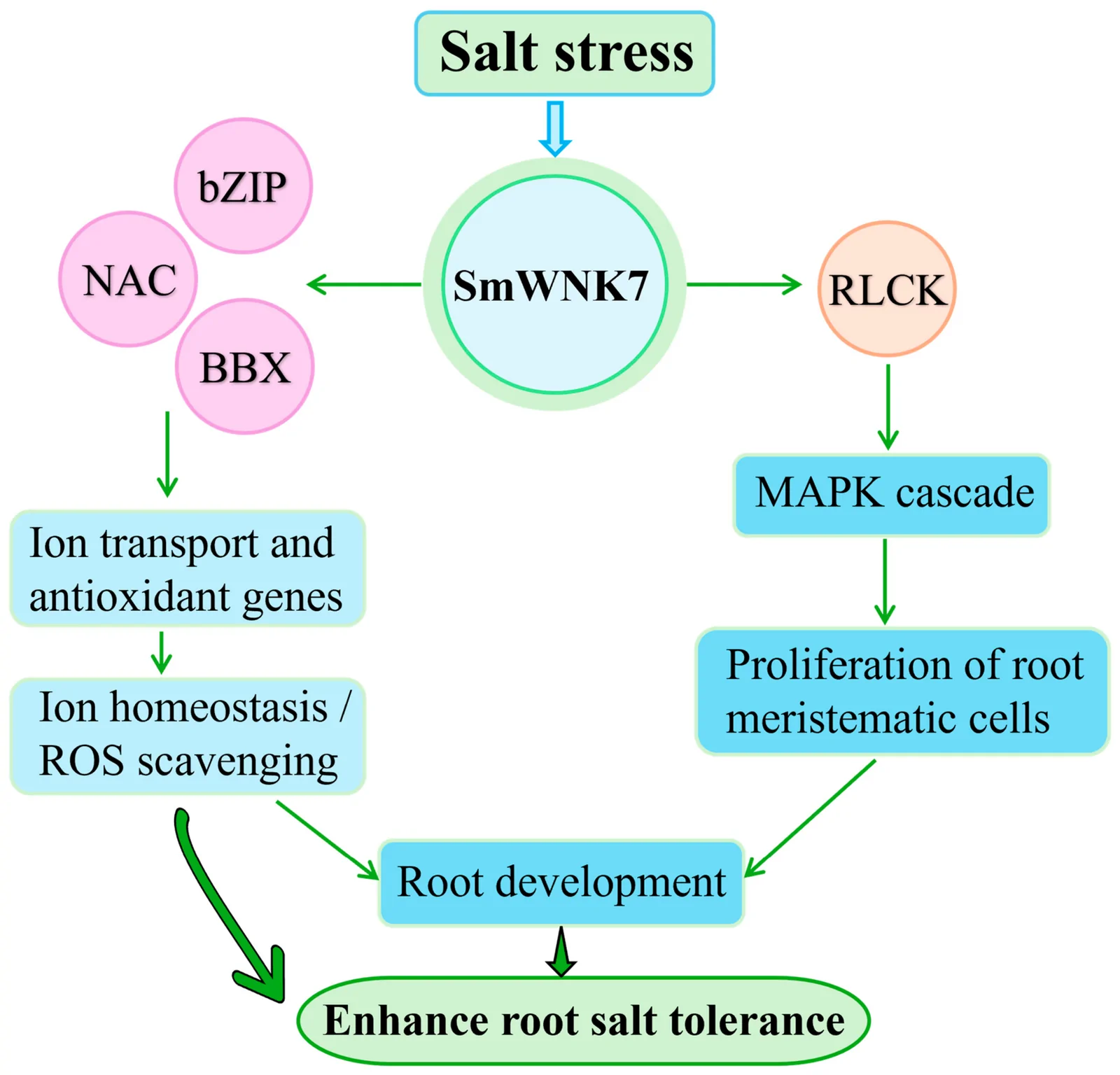
Hypothetical working model diagram of *SmWNK*7 regulating root salt tolerance.

Functional enrichment of 40 interacting proteins enriched ion transport, antioxidant metabolism, hormone signal transduction and MAPK pathway terms. Taken together, we hypothesize a tentative working model illustrating *SmWNK*-mediated salt tolerance in plant roots. Salt stress activates *SmWNK*7 kinase. On the one hand, *SmWNK*7 may phosphorylate bZIP, NAC and BBX transcription factors to induce the expression of ion transport and antioxidant genes. On the other hand, it interacts with RLCK to activate the MAPK cascade signal and regulates meristem cell proliferation. Multiple pathways of signal transduction, ion homeostasis and root development jointly improve salt tolerance (Figure 9).

## 4. Materials and Methods

### 4.1. Genome-Wide Identification and Characterization of the SmWNK Gene Family in Salvia miltiorrhiza

The genome sequence, protein sequence, and annotation files of *S. miltiorrhiza* (accession number: GWHDOEA00000000) were downloaded from the National Center for Bioinformation (CNCB) database [55] (https://www.cncb.ac.cn/). To identify the *SmWNK*s genes in *S. miltiorrhiza*, the amino acid sequences of WNKs from *A. thaliana* (TAIR10-thale cress) and *O. sativa* (V7.0-rice) were downloaded from the Phytozome database [56] (https://phytozome-next.jgi.doe.gov/). These sequences were used as queries to perform local BLASTP (NCBI-BLAST+, version 2.16.0) searches against the *S. miltiorrhiza* protein sequence database (e-value ≤ 1 × 10^−5^). The matched sequences were further filtered, and those with sequence similarity greater than 50% were selected as candidate members of the *SmWNK*s gene family. Finally, the protein sequences of the candidate *SmWNK*s were extracted using Tbtools-II (version 2.303) software [57] and submitted to the NCBI Conserved Domain Database (CDD) [58] (https://www.ncbi.nlm.nih.gov/Structure/bwrpsb/bwrpsb.cgi) (accessed on 8 February 2026) to verify whether they contained the target conserved domains. The ProtParam tool within the ExPASy online database was applied to analyze the number of amino acids, molecular weight, isoelectric point (PI), instability index, aliphatic index, and grand average of hydropathicity (GRAVY) (https://web.expasy.org/protparam/) (accessed on 8 February 2026). Transmembrane domain analysis was performed using the TMHMM-2.0 website [59] (https://services.healthtech.dtu.dk/services/TMHMM-2.0/) (accessed on 8 February 2026). Subcellular localization of *SmWNK*s was predicted using the WoLF PSORT website [60] (https://wolfpsort.hgc.jp/) (accessed on 8 February 2026). Prediction of the protein tertiary structure was carried out using SWISS-MODEL [61].

### 4.2. Sequence Alignment and Phylogenetic Analysis

Phylogenetic analysis was conducted using MEGA11, and a phylogenetic tree was constructed using the Maximum Likelihood (ML) method with 1000 bootstrap replicates. Finally, the resulting phylogenetic tree was visualized and annotated using ChiPlot [62] (https://chiplot.online/) (accessed on 12 February 2026).

Multiple sequence alignment of *SmWNK*s and *AtWNK*s sequences was performed using the ClustalW tool in MEGA11 software. The alignment parameters were set as follows: for pairwise alignment, gap extension penalty: 0.10, gap opening penalty: 10.00; for multiple alignment, gap extension penalty: 0.20, gap opening penalty: 10.00 [63]. GeneDoc software was used to visualize the shading of conserved amino acids [64].

### 4.3. Analysis of Conserved Motifs, Gene Structures and Cis-Acting Elements of SmWNKs

The conserved motifs of *SmWNK* proteins were predicted via the online MEME tool [65] (https://meme-suite.org/meme/tools/meme) (accessed on 15 February 2026) with default parameter settings. The gene structures of *SmWNK*s were obtained from the genome annotation GFF3 file of *S. miltiorrhiza*. The 2 kb upstream sequences of the *SmWNK* genes were extracted, and their cis-acting regulatory elements were predicted using the Plant CARE website [56] (https://bioinformatics.psb.ugent.be/webtools/plantcare/html/) (accessed on 15 February 2026). Finally, TBtools software was used to visualize the conserved motifs, gene structures and cis-acting elements.

### 4.4. Chromosomal Localization and Synteny Analysis of SmWNKs

The “Gene Location Visualize” module of TBtools was used to visualize the chromosomal physical locations of *SmWNK* gene family members. Genome-wide synteny analysis was performed using the One Step MCScanX module in TBtools, a circular collinearity block map was generated using Advanced Circos, and the Multiple Synteny Plot module was used to visualize interspecific synteny results [57].

### 4.5. Plant Growth Conditions and Stress Treatments

Plants were grown under a 16 h light/8 h dark photoperiod at 25 °C. Seedlings exhibiting uniform growth were exposed to salt stress by applying 200 mM NaCl. After treatment, samples were collected at 0, 3, 6, 24, and 48 h, rinsed with purified water, immediately frozen in liquid nitrogen, and stored at −80 °C. Each treatment group contained three biological replicates.

### 4.6. Quantitative Real-Time PCR (qRT-PCR)

qRT-PCR primers were designed based on the coding sequence (CDS) of each *SmWNK* gene (Table S11), and SmActin was selected as the internal reference gene. The qPCR amplification was performed using the Hieff qPCR SYBR Green Master Mix (Yeasen Biotechnology) in a two-step protocol on a QuantStudio 5 real-time PCR system (Thermo Fisher Scientific, Waltham, MA, USA). The relative expression levels of *SmWNK*s were calculated using the 2^−ΔΔCt^ method [66]. Each sample included three biological replicates and three technical replicates. The expression level at 0 h was used as the calibrator (set to 1) to determine the relative fold change at subsequent time points.

### 4.7. Construction of Overexpression Vectors

Total RNA was extracted from *S. miltiorrhiza* roots using the RNAprep Pure Plant Plus Kit (Tiangen Biotech, Beijing, China). First-strand cDNA was synthesized using the Hifair^®^ II 1st Strand cDNA Synthesis SuperMix for qPCR (Yeasen Biotechnology, Shanghai, China). Using the reverse-transcribed cDNA as the template, the target fragment of the *SmWNK*7 gene was amplified by PCR with TransStart^®^ FastPfu DNA Polymerase (TransGen Biotech, Beijing, China). Primer sequences are listed in Table S12.

The overexpression vector pIB360 was linearized using the restriction endonucleases BamHI and NcoI. The linearized vector and the target fragment were then ligated using the ClonExpress^®^ II One Step Cloning Kit (Nanjing Vazyme Biotech Co., Ltd., Nanjing, China). The ligation product was transformed into Escherichia coli competent cells (strain DH5α). Positive clones were preliminarily confirmed by colony PCR amplification (Table S13) and 1% agarose gel electrophoresis, followed by sequencing verification (Figure S9).

### 4.8. Genetic Transformation of Tobacco

Sterilized tobacco seeds were sown on germination medium and cultured at 25 °C under a 16 h light/8 h dark photoperiod for 4–5 weeks. Sterile leaves were cut into small pieces and pre-cultured for 2–3 days. The OE-*SmWNK*7 construct was transformed into Agrobacterium tumefaciens strain GV3101 competent cells. Agrobacterium cells were resuspended to an OD600 of 0.2, and the pre-cultured leaf explants were infected for 10–15 min, blotted dry with filter paper, and then co-cultivated for 48–72 h. Subsequently, the explants were transferred to induction medium for callus induction for approximately 10 days and then moved to selection medium containing the appropriate selective agent and cultured at 25 °C for 15–30 days. Positively selected calli after the second round of selection were inoculated onto differentiation medium and cultured under the same light and temperature conditions for 15–30 days. The differentiated shoots were transferred to rooting/seedling-strengthening medium for 7–10 days. Genomic DNA was extracted using the CTAB method [67] and subjected to PCR analysis (Figures S10 and S11). The primers used for identification of positive plants are listed in Table S14.

### 4.9. Agrobacterium-Mediated Transformation of OE-SmWNK7 and Hairy Root Induction in S. miltiorrhiza

According to the instructions for K599 competent cells (Coolaber, Cat. No. CC410), the cyclic empty vector (pIB_360) and *SmWNK*7 gene were separately transformed into K599 competent cells. Positive clones were identified by bacterial liquid PCR, followed by primer verification and sequencing confirmation (Table S13). The primary cultured Agrobacterium was incubated until its OD600 value reached 1, which was used to infect the base of single leaves of *S. miltiorrhiza*. The petioles were inserted vertically into the soil, and 3–5 mL of Agrobacterium suspension was inoculated on each leaf. Subsequently, the infected leaves were cultured in a climate chamber at 25 °C with a day-night ratio of 16:8. Positive hairy roots were formed after 2 weeks of culture.

### 4.10. Seed Germination and Root Length Test

Wild-type (WT) tobacco and tobacco overexpressing the WNK7 gene (OE-*SmWNK*7) were used as experimental materials. Seeds were surface sterilized by treatment with 75% ethanol for 1 min, followed by 5% sodium hypochlorite for 10 min, and then rinsed five times with sterile water until the wash solution became clear. The sterilized seeds were vernalized in sterile water at 4 °C for 24 h. The vernalized seeds were then sown on 1/2 MS solid medium containing 0, 100, or 200 mM NaCl [68]. After sowing, the seeds were cultured in the dark at 25 °C for 2 days and then transferred to a 25 °C incubator under a 16 h light/8 h dark photoperiod. Seeds were judged as germinated when testa and endosperm rupture and radicle protrusion were observed. The endpoint for germination scoring was defined as the time when WT seeds on 1/2 MS medium no longer germinated [69,70].

For the root length assay, WT and OE-*SmWNK*7 tobacco seeds were placed on 1/2 MS medium. After germination, seedlings were grown for 7 days at 25 °C with a 16 h light/8 h dark photoperiod. The seedlings were then transferred to 1/2 MS medium containing 0, 100, and 200 mM NaCl for vertical culture. The position of the root tip was marked at the time of transfer, and the seedlings were grown for an additional 7 days under the same temperature and photoperiod conditions. Subsequently, the primary root elongation was measured, and the relative inhibition rate was calculated accordingly [71].

### 4.11. Physiological Assays of SmWNK7-Overexpressing Plants Under Salt Stress

Tobacco plants overexpressing *SmWNK*7 and WT plants with similar growth status, comparable plant height, and no visible pests or diseases were selected as experimental materials. Initial growth conditions were strictly controlled (25 °C, 16 h light/8 h dark photoperiod) to ensure uniformity of the experimental material. Three salt stress treatment groups were established, in which plants were irrigated with 0, 100, or 200 mM NaCl solutions, respectively. For each treatment, three biological replicates were included for each genotype. After 24 h of continuous salt stress, healthy leaves from the same position of each plant in each treatment group were collected, rinsed clean, immediately frozen in liquid nitrogen, and stored at −80 °C for subsequent physiological measurements. Physiological indices were determined according to the instructions of the corresponding assay kits from Shanghai Enzyme-linked Biotechnology Co., Ltd. (Shanghai, China) (https://www.mlbio.cn/category-280-b0.html) (accessed on 12 March 2026). Catalase (CAT) activity was measured using the ultraviolet absorption method, peroxidase (POD) activity was determined by the micro-method, malondialdehyde (MDA) content was measured using the micro method, and root activity was assessed by the TTC method.

### 4.12. Yeast Two-Hybrid Assay

The yeast two-hybrid assay was performed using the Y2HGold-GAL4 Yeast Two-Hybrid Library Screening Kit (Coolaber) according to the manufacturer’s instructions. The open reading frame (ORF) of the *SmWNK*7 gene was cloned into the pGBKT7 vector (BD) to construct the bait vector pGBKT7-*SmWNK*7 (BD-*SmWNK*7). After co-transforming the Y2HGold [BD-*SmWNK*7] competent cells with the *S. miltiorrhiza* cDNA library plasmids, the mixture was spread on SD/-Leu/-Trp solid medium and cultured at 30 °C for 3–5 days. The blue single colonies grown were picked to complete the primary screening. The blue single colonies obtained from the primary screening were streaked on SD/-Ade/-His/-Leu/-Trp solid medium (containing X-α-Gal and the optimal AbA concentration) and cultured at 30 °C for 2–4 days to purify the interacting clones. The positive interacting clones were identified by colony PCR and Sanger sequencing verification. Sequences were aligned using the CNCB BLAST (version 2.16.0) tool. By integrating *S. miltiorrhiza* cDNA library data, positive clones were identified to screen downstream interacting proteins of *SmWNK*7.

## 5. Conclusions

In summary, we systematically characterized nine *SmWNK* genes in *S. miltiorrhiza*, which were divided into four groups based on phylogenetic relationships. The gene structures and protein motifs were relatively conserved within each group. qRT-PCR analysis revealed that all *SmWNK* genes except *SmWNK*3 were significantly affected by salt stress. Heterologous overexpression of *SmWNK*7 in tobacco increased CAT and POD activities, reduced MDA content, and alleviated membrane lipid peroxidation damage, thereby promoting plant growth and enhancing salt stress tolerance. We further performed yeast two-hybrid screening and obtained 40 potential interacting partners of *SmWNK*7. Six candidates belong to transcription factor families, and one encodes a protein kinase. Functional annotation revealed that these 40 proteins were assigned to 81 GO terms and 27 KEGG pathways. This study provides valuable insights into the molecular mechanisms of WNK genes, and *SmWNK*7 is a promising candidate gene for future validation and breeding studies in *S. miltiorrhiza*.

## Figures and Tables

**Table 1:** Detailed information about the members of the *Salvia miltiorrhiza* WNK gene family.

Gene	Gene ID	Number of Amino Acids	Molecular Weight	Theoretical pI	Instability Index	Aliphatic Index	Grand Average of Hydropathicity	Subcellular Localization Prediction
*SmWNK*1	GWHGDOEA004603	629	70,564.05	5.16	42.68	78.44	−0.561	cell nucleus
*SmWNK*2	GWHGDOEA010072	299	33,874.33	5.69	39.97	83.48	−0.404	cytoplasm
*SmWNK*3	GWHGDOEA014169	590	66,890.4	5.74	61.09	70.41	−0.625	cell nucleus
*SmWNK*4	GWHGDOEA014827	622	70,427.43	4.92	49.63	83.54	−0.612	chloroplast
*SmWNK*5	GWHGDOEA018168	295	33,749.37	5.53	35.45	86.88	−0.472	cytoplasm
*SmWNK*6	GWHGDOEA023413	723	81,630	4.97	45.73	77.8	−0.506	cell nucleus
*SmWNK*7	GWHGDOEA023883	602	68,586.2	6.02	42.49	78.87	−0.558	cell nucleus
*SmWNK*8	GWHGDOEA027985	511	58,350.45	4.78	45.13	80.47	−0.459	cell nucleus
*SmWNK*9	GWHGDOEA029002	680	76,437.25	5.72	45.94	75.74	−0.489	chloroplast

## Data Availability

All data supporting the findings of this study are available within the paper and its Supplementary Information.

## Supplementary Materials

The following supporting information can be downloaded at https://www.mdpi.com/article/10.3390/plants15162438/s1, Supplementary File S1: Sanger-sequencing chromatograms of 40 candidate interacting proteins of *SmWNK*7 screened via yeast-two-hybrid assay; Figure S1: Transmembrane domain prediction of *SmWNK*s; Figure S2: Three-dimensional structure prediction of *S. miltiorrhiza SmWNK*s, grouped by their phylogenetic relationships; Figure S3: Multiple amino acid sequence alignment of WNKs from *S. miltiorrhiza* and *A. thaliana*; Figure S4: Relative expression levels of *SmWNK*7 in wild-type (CK) and three independent *SmWNK*7-overexpressing transgenic tobacco lines (OE1, OE2, OE3) detected by qRT-PCR; Figure S5: Statistical graph of total length of hairy roots induced from single leaves of *S. miltiorrhiza* by Agrobacterium infection; Figure S6: Sequencing verification of yeast two-hybrid bait vector BD-*SmWNK*7; Figure S7: Screening and functional enrichment analysis of *SmWNK*7-interacting proteins; Figure S8: Agarose gel electrophoresis confirming bacterial liquid PCR products of candidate interacting proteins downstream of *SmWNK*7; Figure S9: Sequencing verification of recombinant pIB360-*SmWNK*7 positive clones; Figure S10: Workflow of Agrobacterium leaf disc transformation for *SmWNK*7-overexpressing tobacco lines; Figure S11: Agarose gel electrophoresis identification of positive *SmWNK*7-overexpressing tobacco lines; Table S1: The protein sequences of the predicted WNKs from the *Salvia miltiorrhiza* genome; Table S2: Accession numbers of WNK genes used for phylogenetic tree construction; Table S3: Collinear genes among different species; Table S4: The promoter cis-acting elements of *SmWNK* genes; Table S5: Summary of the number of cis-acting element types of *SmWNK*s; Table S6: Statistical table of total hairy root length for each line in single-leaf agrobacterium infection assay of *Salvia miltiorrhiza*; Table S7: PCR primer sequences for identification of recombinant vector (BD-*SmWNK*7); Table S8: Primer sequences for bacterial liquid PCR of positive clones in yeast two-hybrid screening; Table S9: GO enrichment analysis of candidate interacting proteins identified by yeast two-hybrid screening; Table S10: KEGG enrichment analysis of candidate interacting proteins identified by yeast two-hybrid screening; Table S11: The primer sequences of qPCR in this project; Table S12: The primer sequences of gene overexpression used in this project; Table S13: Primers for PCR of OE-*SmWNK*7 positive bacterial liquid; Table S14: The primer sequences for identification of positive plants (OE-*SmWNK*7) used in this experiment.
