# Supplementary material for: Identification of WNK Gene in Salvia miltiorrhiza Reveals SmWNK7 Positively Regulates Root Growth and Salt Tolerance
Source: Plants (Basel). 2026 Aug 11;15(16):2438. doi: 10.3390/plants15162438 (PMC13516755; doi:10.3390/plants15162438)
Supplement: Supplementary file 1 [file plants-15-02438-s001.zip › Supplementary figures.pdf]

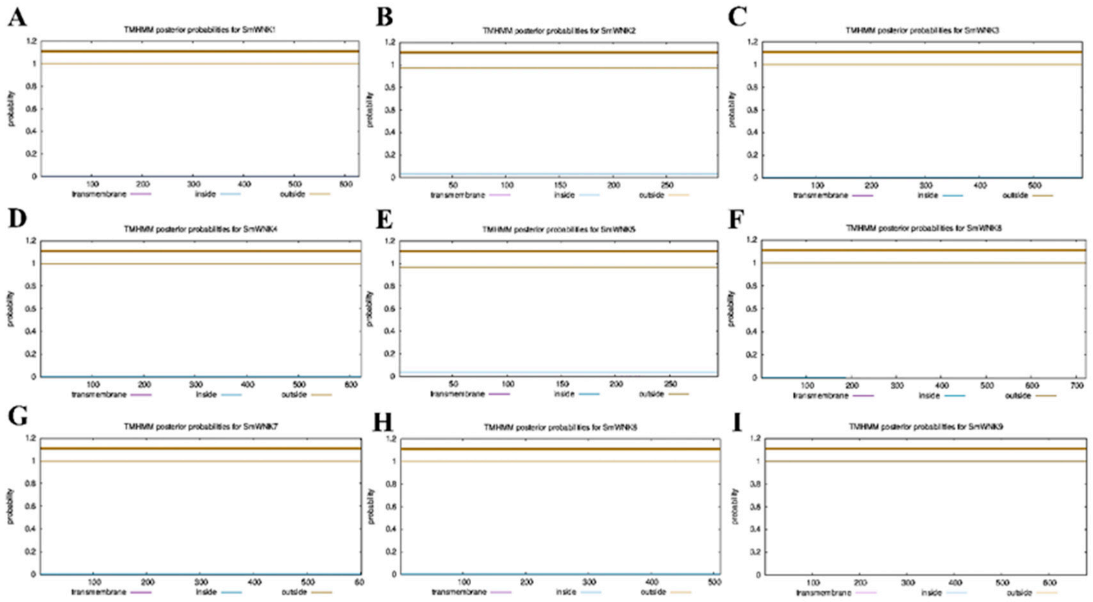

Figure S1. Transmembrane domain prediction of *SmWNK*s.

(**A**) *SmWNK*1; (**B**) *SmWNK*2; (**C**) *SmWNK*3; (**D**)*SmWNK*4; (**E**)*SmWNK*5; (**F**) *SmWNK*6; (**G**) *SmWNK*7; (**H**) *SmWNK*8; (**I**) *SmWNK*9. Inside represents the intracellular region, and the higher the inside value, the higher the probability that the amino acid is located in the intracellular region. Outside indicates the extracellular region, and a higher value of outside indicates that the amino acid is more likely to be located in the extracellular region. Transmembrane means transmembrane region.

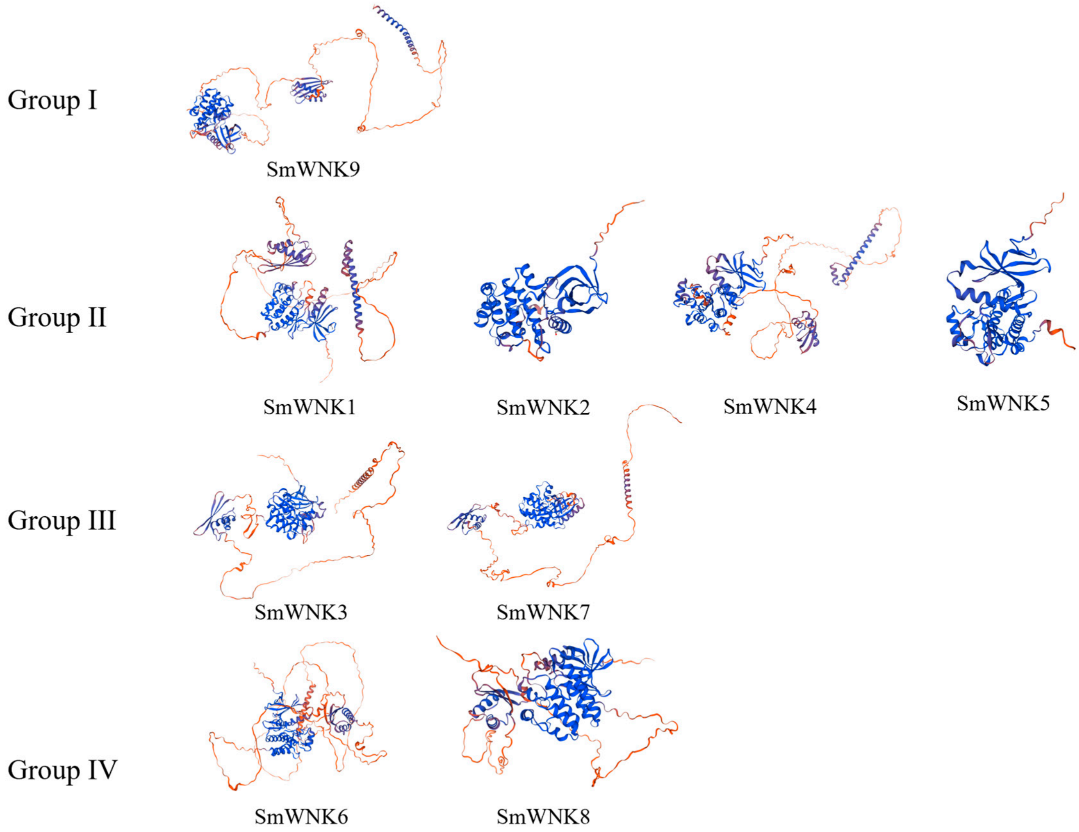

Figure S2. Three-dimensional structure prediction of *S. miltiorrhiza SmWNK*s, grouped by their phylogenetic relationships.

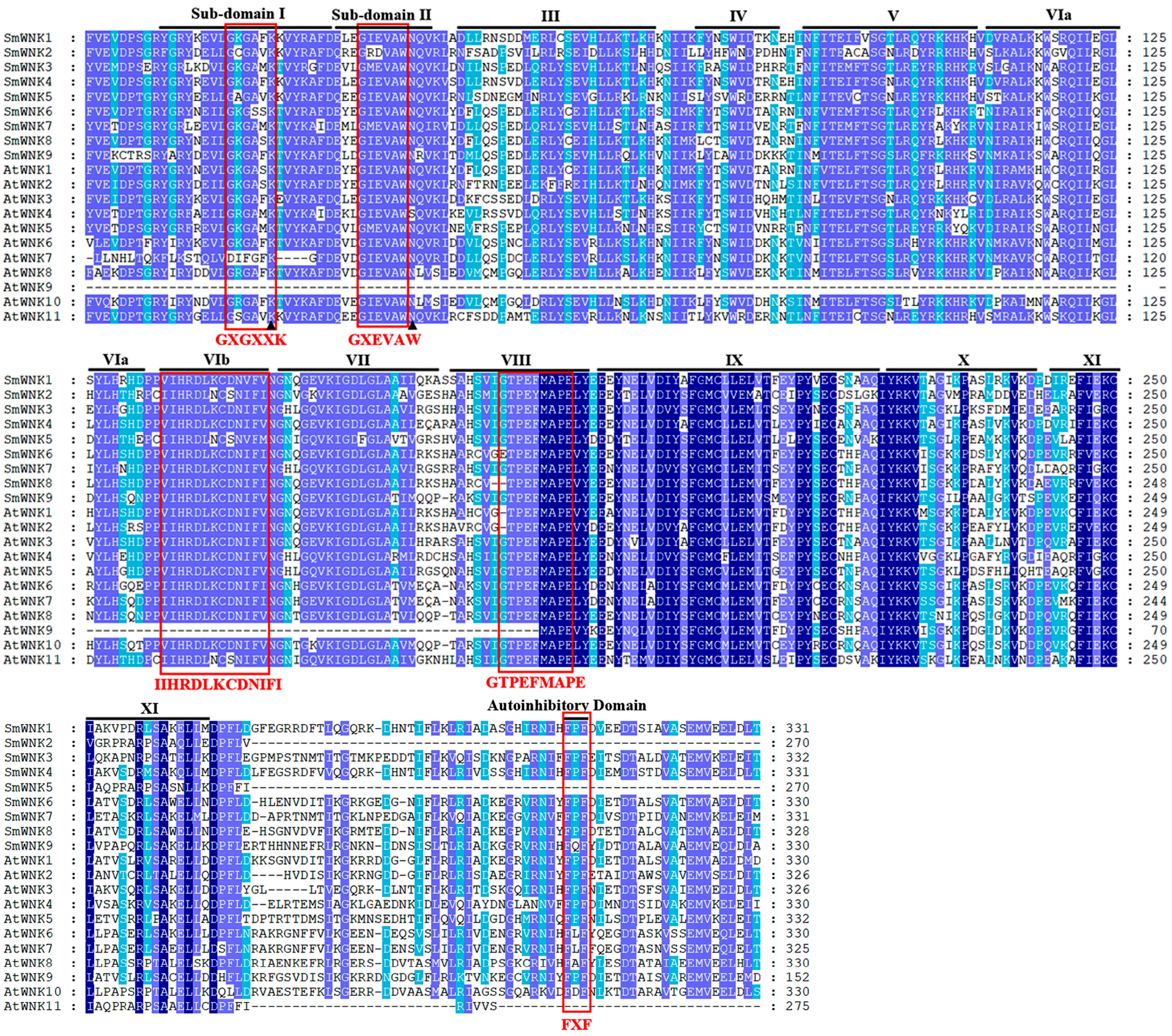

Figure S3. Multiple amino acid sequence alignment of WNKs from *S. miltiorrhiza* and *A. thaliana*. The conserved domains and motifs were highlighted. Shading of amino acids indicates degree of con-servation: purple-90%-100% amino acid conservation; lilac-80%-90% amino acid conservation; blue-70%-80% amino acid conservation. Amino acid position for individual genes is marked on the right.

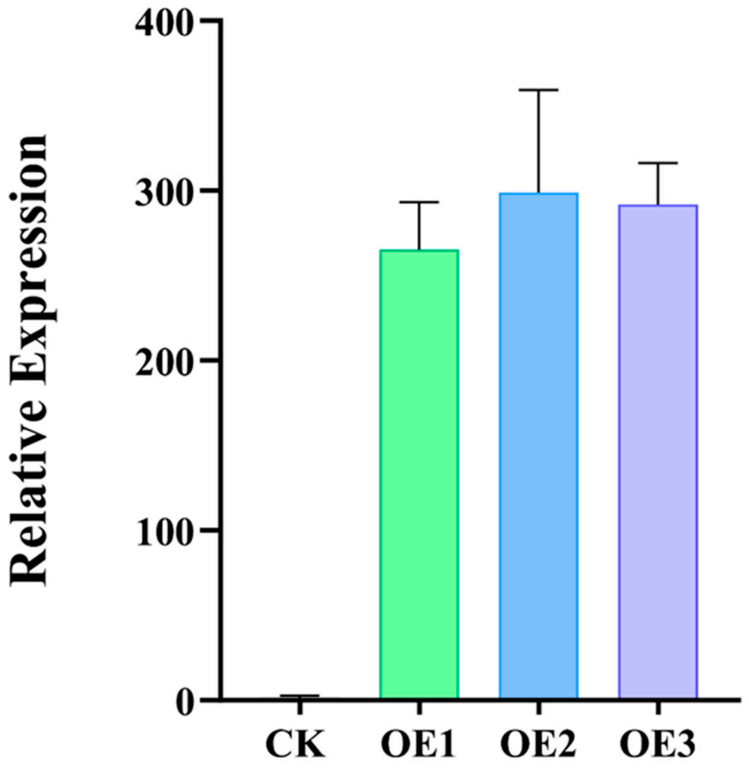

Figure S4. Relative expression levels of *SmWNK7* in wild-type (CK) and three independent *SmWNK7*-overexpressing transgenic tobacco lines (OE1, OE2, OE3) detected by qRT-PCR. The $2^{-\Delta\Delta CT}$ method was used for relative quantification. The values are expressed as the mean ± standard deviation (SD) of three replicates.

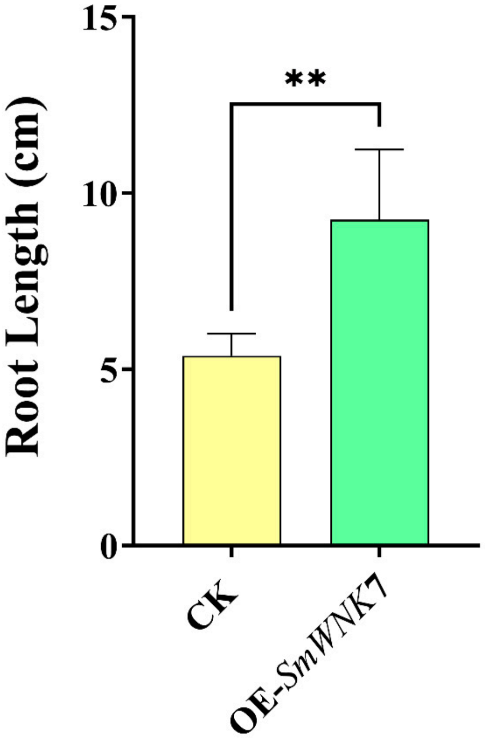

Figure S5. Statistical graph of total length of hairy roots induced from single leaves of *S. miltiorrhiza* by *Agrobacterium* infection. Student's t-test was used for data analysis (n = 4). Asterisks indicate significant differences between OE-*SmWNK*7 and CK wild-type plants (*P < 0.05; **P < 0.01; ***P < 0.001).

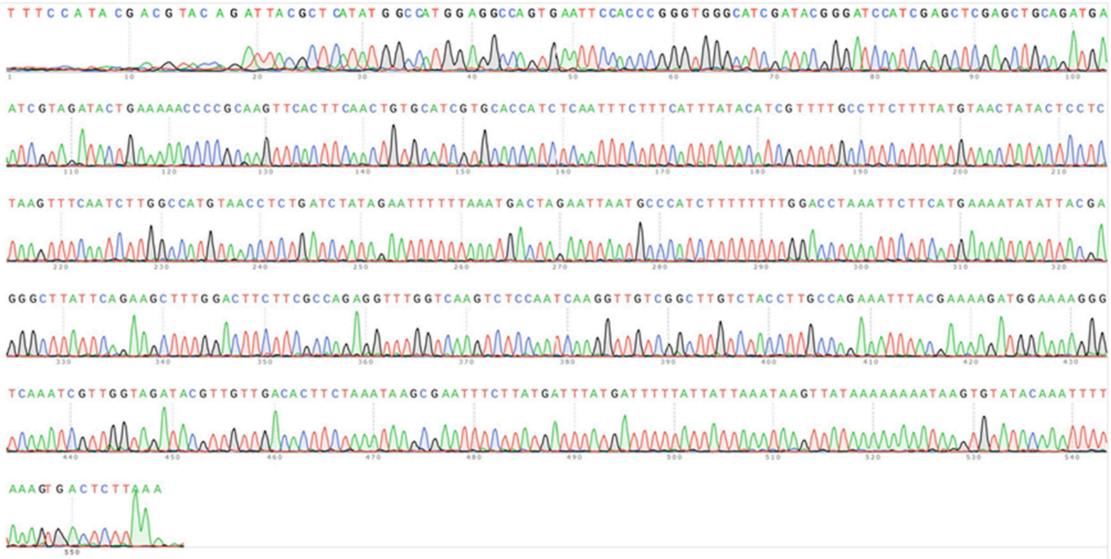

Figure S6. Sequencing verification of yeast two-hybrid bait vector BD-*SmWNK*7.

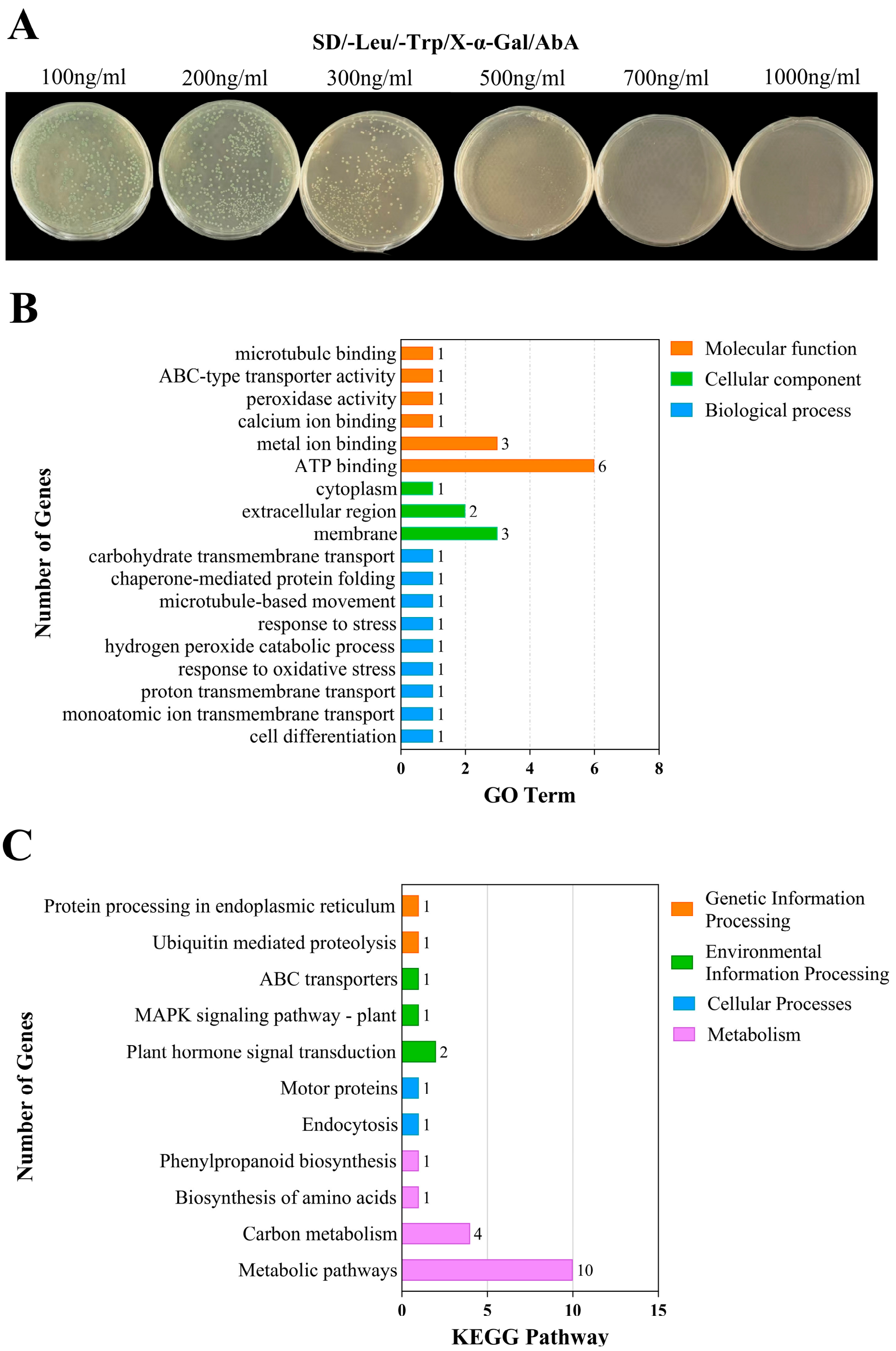

FigureS7. Screening and functional enrichment analysis of *SmWNK7*-interacting proteins. (A) On SD/-Leu/-Trp/X- α -Gal/AbA screening media containing different AbA concen-trations, the optimal AbA concentration for yeast hybridization of the BD-*SmWNK7* bait protein was determined; (B) GO classification histogram; (C) KEGG classification histogram. The vertical axis shows the names of pathways or functional terms, and the horizontal axis indicates the number of genes assigned to each corresponding entry. The legend on the right denotes the primary classification of each pathway or term.

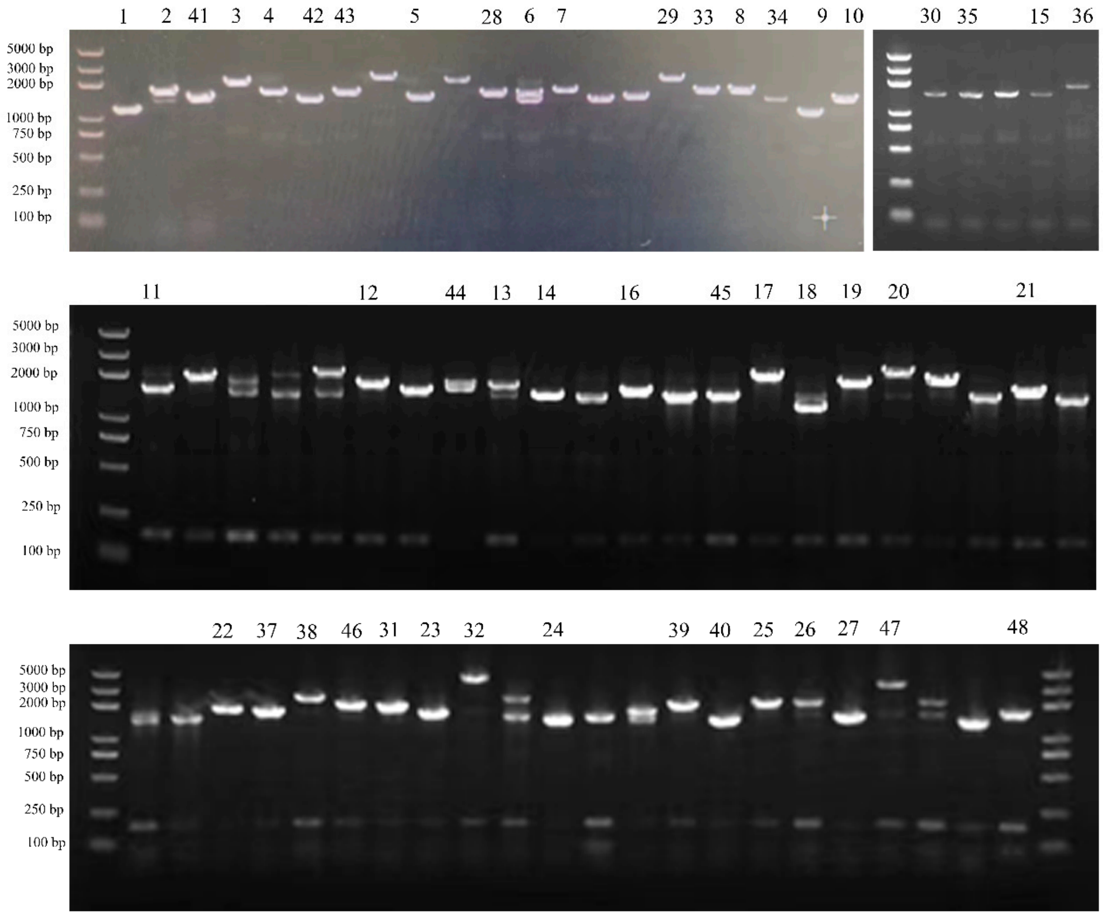

Figure S8. Agarose gel electrophoresis confirming bacterial liquid PCR products of candidate interacting proteins downstream of *SmWNK7*. These numbers correspond to candidate interacting proteins obtained from preliminary screening before redundancy removal.

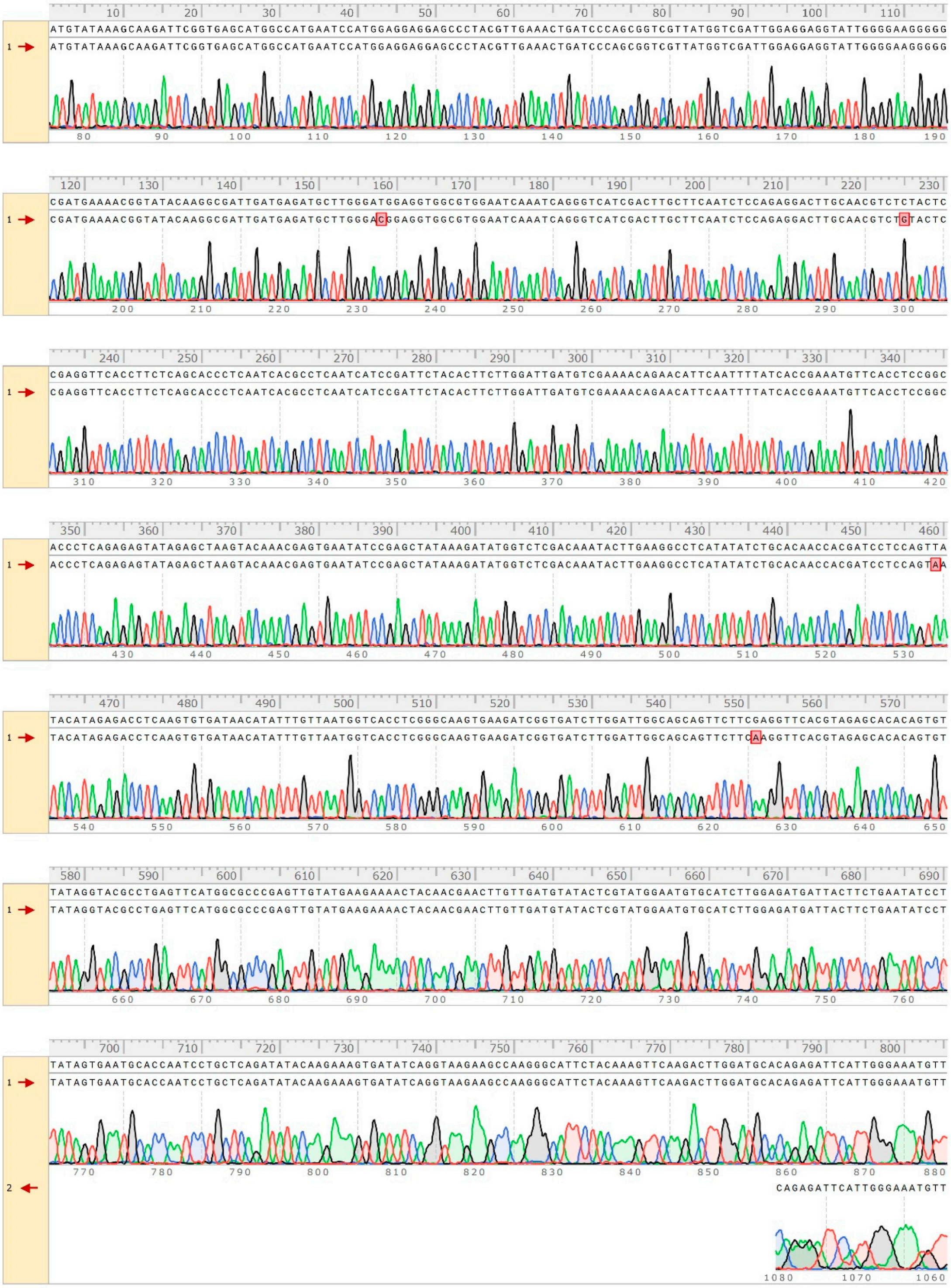
ATGTATAAAGCAAGATTCGGTGAGCATGGCCATGAATCCATGGAGGAGGAGCCCTACGTTGAAACTGATCCCAGCGGTCGTTATGGTCGATTGGAGGAGGTATTGGGGAAGGGGG
ATGTATAAAGCAAGATTCGGTGAGCATGGCCATGAATCCATGGAGGAGGAGCCCTACGTTGAAACTGATCCCAGCGGTCGTTATGGTCGATTGGAGGAGGTATTGGGGAAGGGGG
CGATGAAAACGGTATACAAGGCGATTGATGAGATGCTTGGGATGGAGGTGGCGTGGAATCAAATCAGGGTCATCGACTTGCTTCAATCTCCAGAGGACTTGCAACGTCTCTACTC
CGATGAAAACGGTATACAAGGCGATTGATGAGATGCTTGGGACGGAGGTGGCGTGGAATCAAATCAGGGTCATCGACTTGCTTCAATCTCCAGAGGACTTGCAACGTCTGTACTC
CGAGGTTCACCTTCTCAGCACCCTCAATCACGCCTCAATCATCCGATTCTACACTTCTTGGATTGATGTCGAAAACAGAACATTCAATTTTATCACCGAAATGTTCACCTCCGGC
CGAGGTTCACCTTCTCAGCACCCTCAATCACGCCTCAATCATCCGATTCTACACTTCTTGGATTGATGTCGAAAACAGAACATTCAATTTTATCACCGAAATGTTCACCTCCGGC
ACCCTCAGAGAGTATAGAGCTAAGTACAAACGAGTGAATATCCGAGCTATAAAGATATGGTCTCGACAAATACTTGAAGGCCTCATATATCTGCACAACCACGATCCTCCAGTTA
ACCCTCAGAGAGTATAGAGCTAAGTACAAACGAGTGAATATCCGAGCTATAAAGATATGGTCTCGACAAATACTTGAAGGCCTCATATATCTGCACAACCACGATCCTCCAGTAA
TACATAGAGACCTCAAGTGTGATAACATATTTGTTAATGGTCACCTCGGGCAAGTGAAGATCGGTGATCTTGGATTGGCAGCAGTTCTTCGAGGTTCACGTAGAGCACACAGTGT
TACATAGAGACCTCAAGTGTGATAACATATTTGTTAATGGTCACCTCGGGCAAGTGAAGATCGGTGATCTTGGATTGGCAGCAGTTCTTCAAGGTTCACGTAGAGCACACAGTGT
TATAGGTACGCCTGAGTTCATGGCGCCCGAGTTGTATGAAGAAAACTACAACGAACTTGTTGATGTATACTCGTATGGAATGTGCATCTTGGAGATGATTACTTCTGAATATCCT
TATAGGTACGCCTGAGTTCATGGCGCCCGAGTTGTATGAAGAAAACTACAACGAACTTGTTGATGTATACTCGTATGGAATGTGCATCTTGGAGATGATTACTTCTGAATATCCT
TATAGTGAATGCACCAATCCTGCTCAGATATACAAGAAAGTGATATCAGGTAAGAAGCCAAGGGCATTCTACAAAGTTCAAGACTTGGATGCACAGAGATTCATTGGGAAATGTT
TATAGTGAATGCACCAATCCTGCTCAGATATACAAGAAAGTGATATCAGGTAAGAAGCCAAGGGCATTCTACAAAGTTCAAGACTTGGATGCACAGAGATTCATTGGGAAATGTT
CAGAGATTCATTGGGAAATGTT

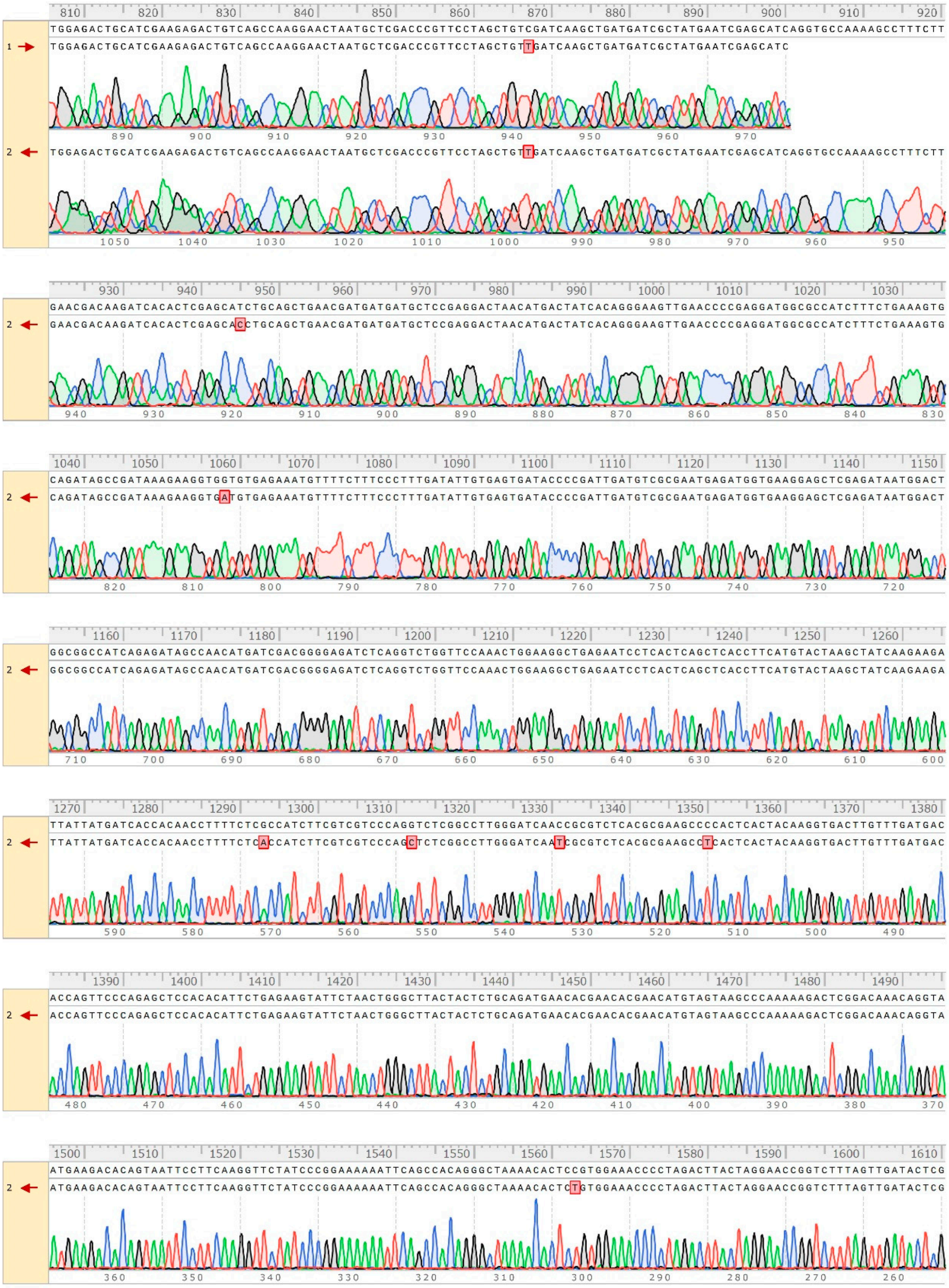

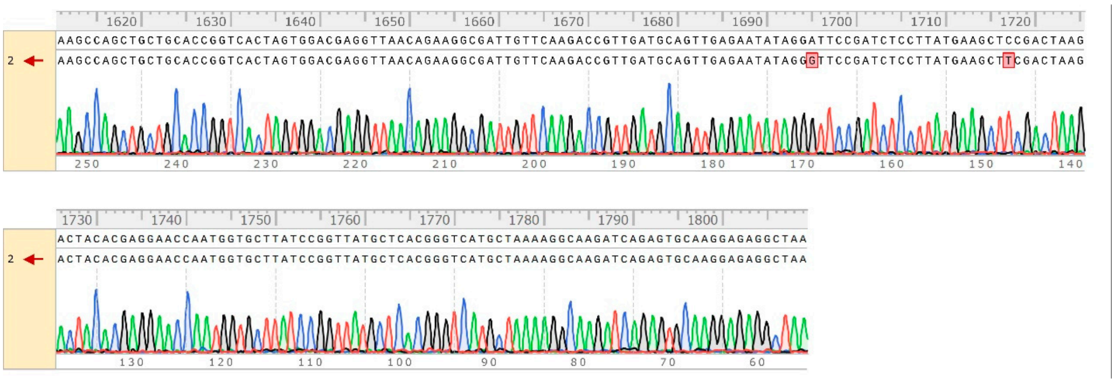

Figure S9. Sequencing verification of recombinant pIB360-*SmWNK7* positive clones.

The base sequence above represents the sequence from reference genome, and the numbers above it represent the base positions. The base sequence below represents the Sanger sequencing result of gene cloning, and the bottom represents the chromatogram of Sanger sequencing.

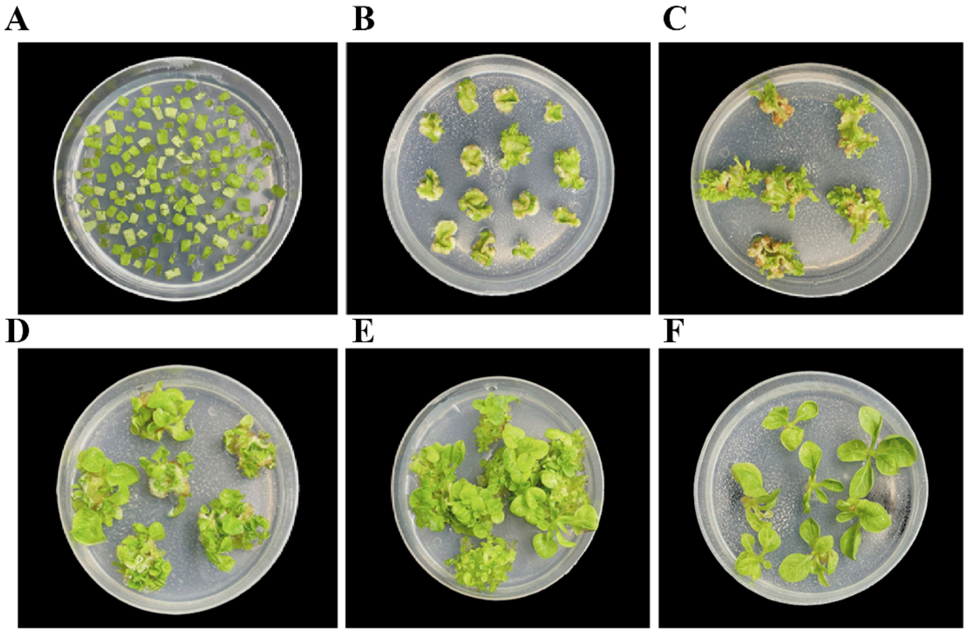

Figure S10. Workflow of Agrobacterium leaf disc transformation for *SmWNK7*-overexpressing tobacco lines.

(**A**) Co-cultivation; (**B**) Callus formation; (**C**) Initial selection; (**D**) Second selection; (**E**) Shoot differentiation; (**F**) Root induction.

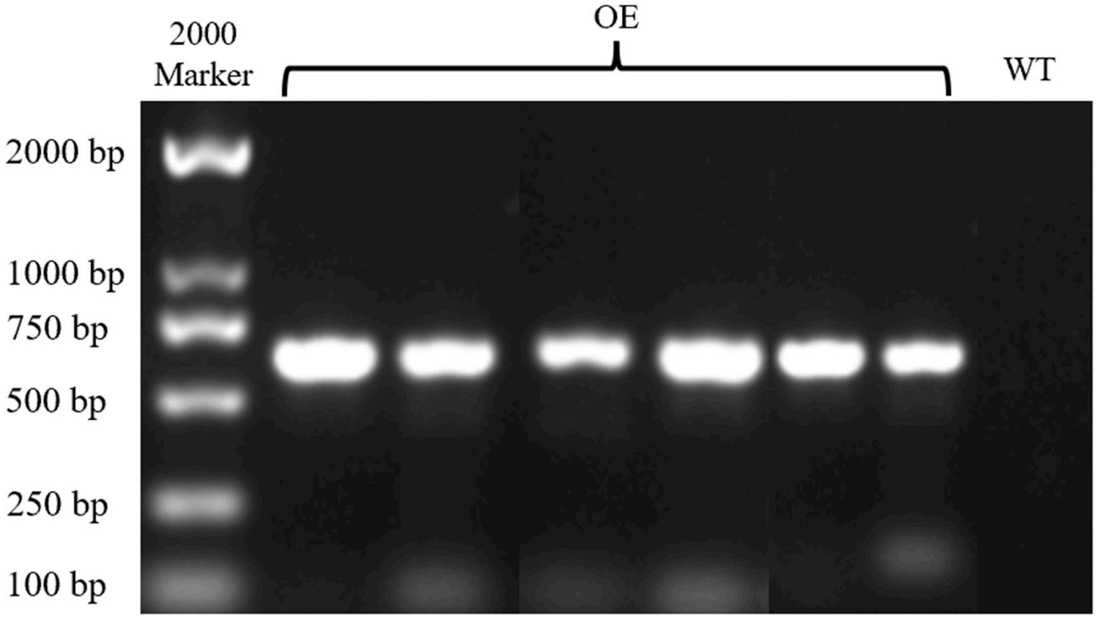

Figure S11. Agarose gel electrophoresis identification of positive *SmWNK7*-overexpressing tobacco lines.
