# Supplementary material for: Identification of WNK Gene in Salvia miltiorrhiza Reveals SmWNK7 Positively Regulates Root Growth and Salt Tolerance
Source: Plants (Basel). 2026 Aug 11;15(16):2438. doi: 10.3390/plants15162438 (PMC13516755; doi:10.3390/plants15162438)
Supplement: Supplementary file 1 [file plants-15-02438-s001.zip › Supplementary File S1/Supplementary file3/gene 11.pdf]

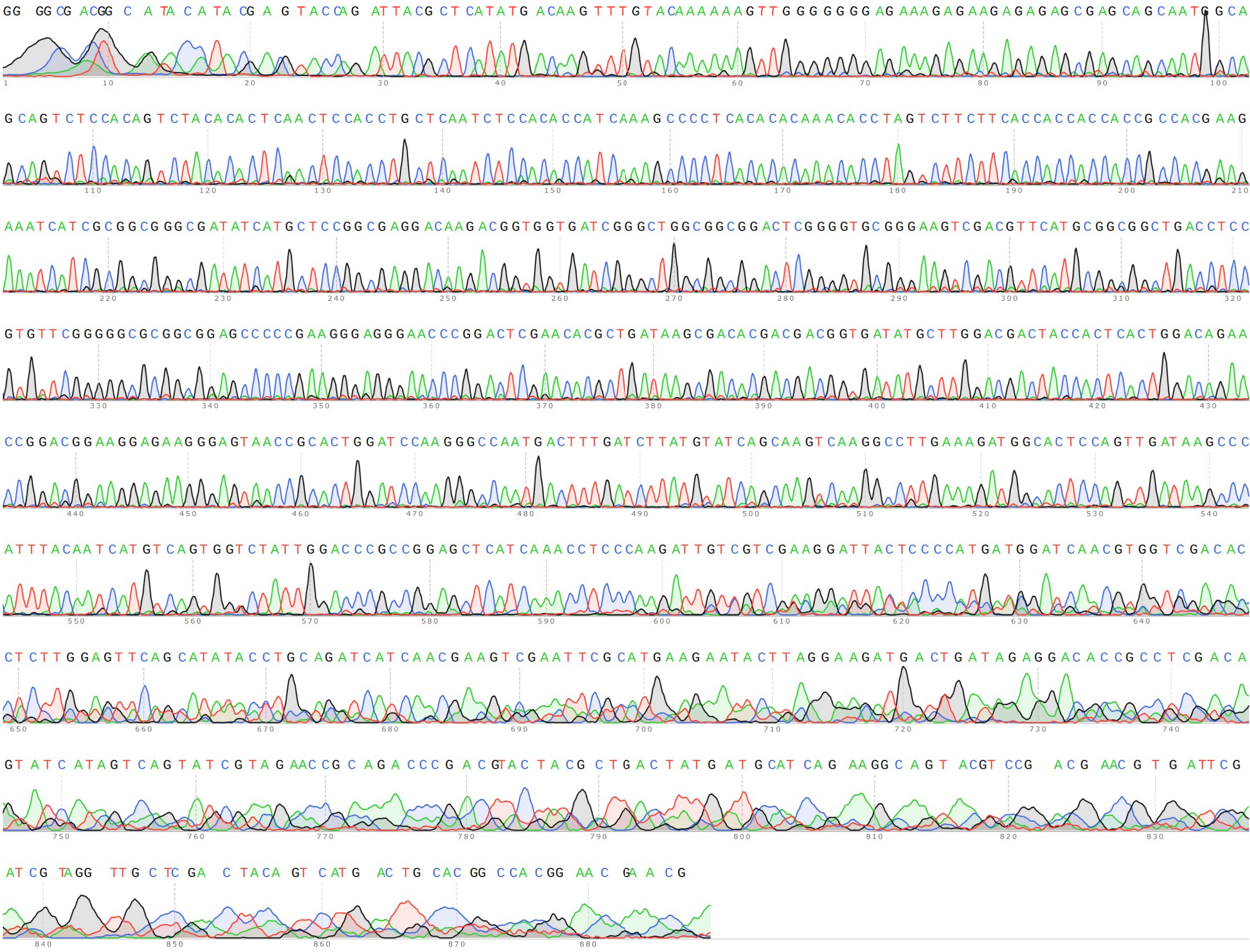
GG GGCG ACGG C A TA C A TA CG A G TACCAG ATTACGCTCATATG ACAAG TTTGTACAAAAAAGTTGGGGGGAGAAAGAGAAGAGAGAGCGAGCAGCAATGGCA
GCAGTCTCCACAGTCTACACACTCAACTCCACCTGCTCAATCTCCACACCATCAAAGCCCCTCACACACAAACACCTAGTCTTCTTCACCACCACCACCGCCACGAAG
AAATCATCGCGGCGGGCGATATCATGCTCCGGCGAGGACAAGACGGTGGTGATCGGGCTGGCGGCGGACTCGGGGTGCGGGAAGTCGACGTTCATGCGGCGGCTGACCTCC
GTGTTCGGGGGCGCGGCGGAGCCCCCGAAGGGAGGGAACCCGGACTCGAACACGCTGATAAGCGACACGACGACGGTGATATGCTTGGACGACTACCACTCACTGGACAGAA
CCGGACGGAAGGAGAAGGGAGTAACCGCACTGGATCCAAGGGCCAATGACTTTGATCTTATGTATCAGCAAGTCAAGGCCTTGAAAGATGGCACTCCAGTTGATAAGCCC
ATTTACAATCATGTCAGTGGTCTATTGGACCCGCCGGAGCTCATCAAACCTCCCAAGATTGTCGTCGAAGGATTACTCCCCATGATGGATCAACGTGGTCGACAC
CTCTTGGAGTTCAGCATATACCTGCAGATCATCAACGAAGTCGAATTCGCATGAAGAATACTTAGGAAGATGACTGATAGAGGACACCGCCTCGACA
GTATCATAGTCAGTATCGTAGAACCGCAGACCCGACGTACTACGCTGACTATGATGCATCAGAAGGCAGTACGTCCG ACG AACGTGATTCG
ATCG TAGG TTGCTCGA CTACA GT CATG ACTG CAC GG CCACGG AAC GAACG
