# Supplementary material for: Identification of WNK Gene in Salvia miltiorrhiza Reveals SmWNK7 Positively Regulates Root Growth and Salt Tolerance
Source: Plants (Basel). 2026 Aug 11;15(16):2438. doi: 10.3390/plants15162438 (PMC13516755; doi:10.3390/plants15162438)
Supplement: Supplementary file 1 [file plants-15-02438-s001.zip › Supplementary File S1/Supplementary file3/gene 12.pdf]

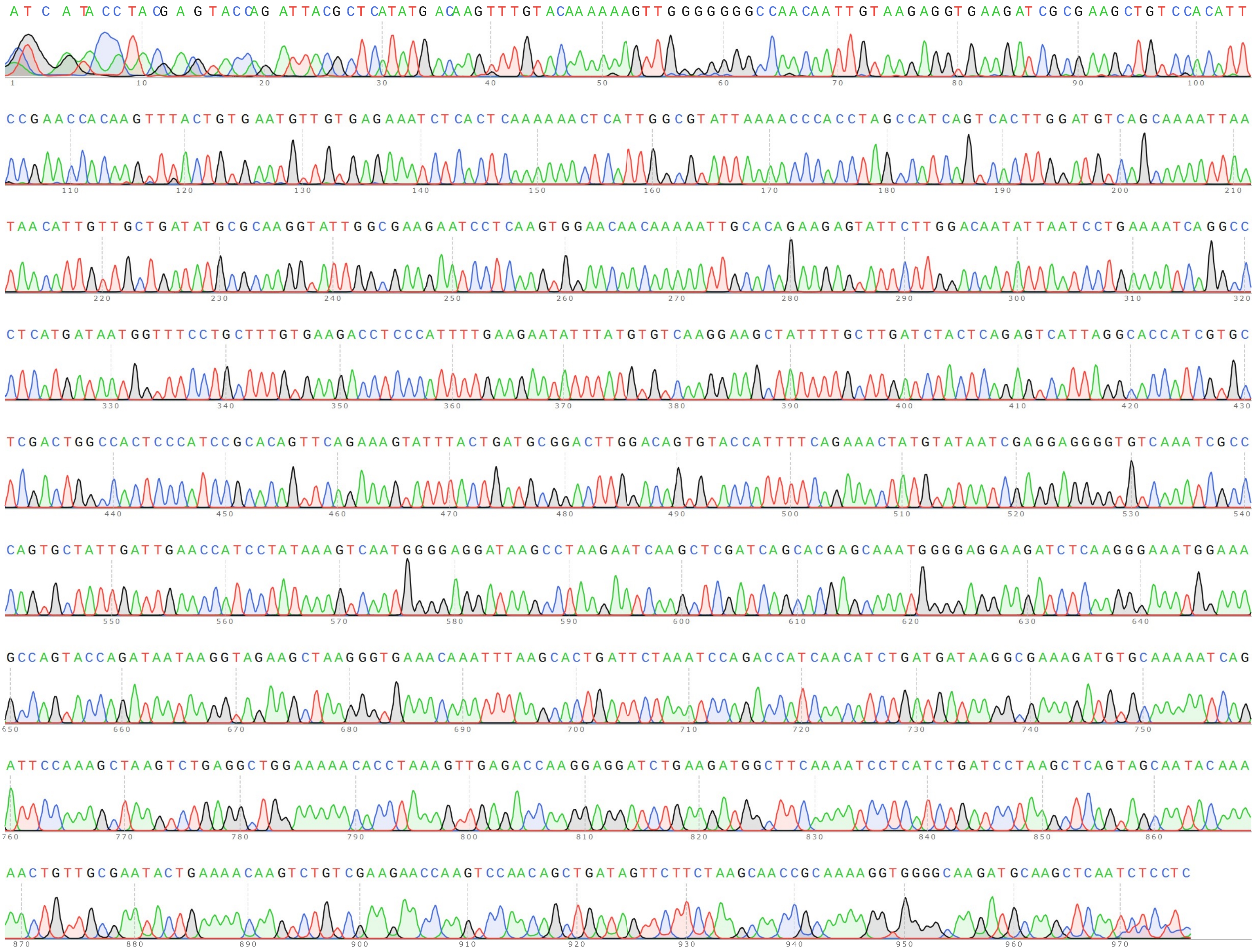
ATCATACCTACGAGTACCAGATTACGCTCATATGACAAGTTTGTACAAAAAAGTTGGGGGGCCAACAATTGTAAGAGGTGAAGATCGCGAAGCTGTCCACATT
CCGAACCACAAGTTTACTGTGAATGTTGTGAGAAATCTCACTCAAAAAACTCATTGGCGTATTAAAACCCACCTAGCCATCAGTCACTTGGATGTCAGCAAAATTAA
TAACATTGTTGCTGATATGCGCAAGGTATTGGCGAAGAATCCTCAAGTGGAACAACAAAAATTGCACAGAAGAGTATTCTTGGACAATATTAATCCTGAAAATCAGGCC
CTCATGATAATGGTTTCCTGCTTTGTGAAGACCTCCCATTTTGAAGAATATTTATGTGTCAAGGAAGCTATTTTGCTTGATCTACTCAGAGTCATTAGGCACCATCGTGC
TCGACTGGCCACTCCCATCCGCACAGTTCAGAAAGTATTTACTGATGCGGACTTGGACAGTGTACCATTTTCAGAAACTATGTATAATCGAGGAGGGGTGTCAAATCGCC
CAGTGCTATTGATTGAACCATCCTATAAAGTCAATGGGGAGGATAAGCCTAAGAATCAAGCTCGATCAGCACGAGCAAATGGGGAGGAAGATCTCAAGGGAAATGGAAA
GCCAGTACCAGATAATAAGGTAGAAGCTAAGGGTGAAACAAATTTAAGCACTGATTCTAAATCCAGACCATCAACATCTGATGATAAGGCGAAAGATGTGCAAAAATCAG
ATTCCAAAGCTAAGTCTGAGGCTGGAAAAACACCTAAAGTTGAGACCAAGGAGGATCTGAAGATGGCTTCAAAATCCTCATCTGATCCTAAGCTCAGTAGCAATACAAA
AACTGTTGCGAATACTGAAAACAAGTCTGTCGAAGAACCAAGTCCAACAGCTGATAGTTCTTCTAAGCAACCGCAAAAGGTGGGGCAAGATGCAAGCTCAATCTCCTC
