# Supplementary material for: Identification of WNK Gene in Salvia miltiorrhiza Reveals SmWNK7 Positively Regulates Root Growth and Salt Tolerance
Source: Plants (Basel). 2026 Aug 11;15(16):2438. doi: 10.3390/plants15162438 (PMC13516755; doi:10.3390/plants15162438)
Supplement: Supplementary file 1 [file plants-15-02438-s001.zip › Supplementary File S1/Supplementary file3/gene 13.pdf]

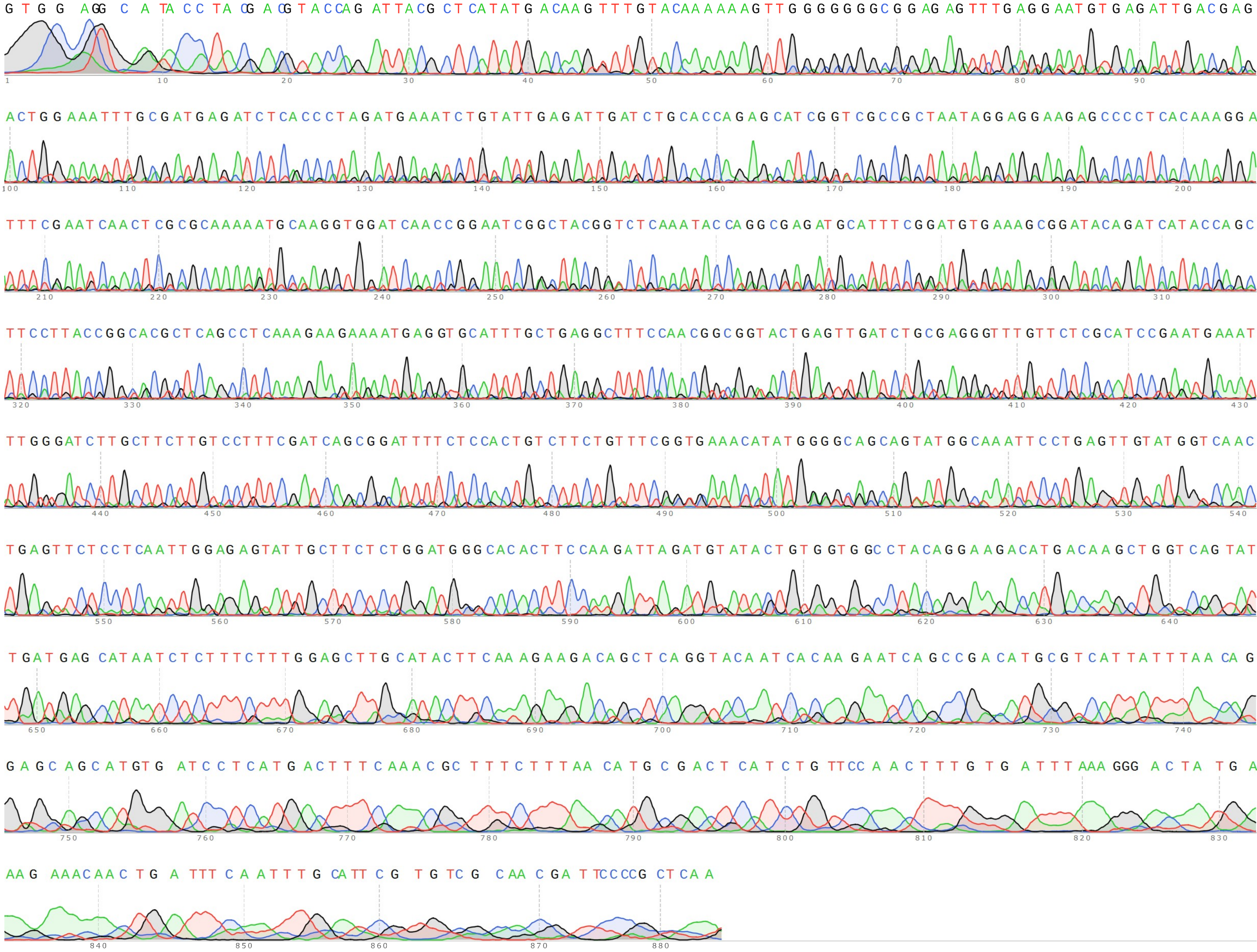
G T G G AGG C A TA C C TA CG A CG T A C CA G ATTACG CT CATATG ACAAG TTTGTACAAAAAAGTTGGGGGGCGGAG AGTTTGAGGAATGTGAGATTGACGAG
ACTGGAAATTTGCGATGAGATCTCACCCTAGATGAAATCTGTATTGAGATTGATCTGCACCAGAGCATCGGTCGCCGCTAATAGGAGGAAGAGCCCCTCACAAAGGA
TTTCGAATCAACTCGCGCAAAAATGCAAGGTGGATCAACCGGAATCGGCTACGGTCTCAAATACCAGGCGAGATGCATTTCGGATGTGAAAGCGGATACAGATCATACCAGC
TTCCTTACCGGCACGCTCAGCCTCAAAGAAGAAAATGAGGTGCATTTGCTGAGGCTTTCCAACGGCGGTACTGAGTTGATCTGCGAGGGTTTGTTCTCGCATCCGAATGAAAT
TTGGATCTTGCTTCTTGTCCTTTCGATCAGCGGATTTTCTCCACTGTCTTCTGTTTCGGTGAAACATATGGGGCAGCAGTATGGCAAATTCCTGAGTTGTATGGTCAAC
TGAGTTCTCCTCAATTGGAGAGTATTGCTTCTCTGGATGGGCACACTTCCAAGATTAGATGTATACTGTGGTGGCCTACAGGAAGACATGACAAGCTGGTCAGTAT
TGATGAG CATAATCTCTTTCTTTGGAGCTTGCATACTTCAAAGAAGACAGCTCAGGTACAATCACAAGAATCAGCCGACATGCGTCATTATTTAACAG
GAGCAGCATGTG ATCCTCATGACTTTCAAACGC TTTCTTTAA CATGCGACT CATCTG TTCCAACTTTG TG ATTTAAAGGG ACTA TGA
AAG AAACAACTG A TTT CA ATTTG CATT CG TGTCG CAA CGA TTCCCCG CTCAA
