# Supplementary material for: Identification of WNK Gene in Salvia miltiorrhiza Reveals SmWNK7 Positively Regulates Root Growth and Salt Tolerance
Source: Plants (Basel). 2026 Aug 11;15(16):2438. doi: 10.3390/plants15162438 (PMC13516755; doi:10.3390/plants15162438)
Supplement: Supplementary file 1 [file plants-15-02438-s001.zip › Supplementary File S1/Supplementary file3/gene 33.pdf]

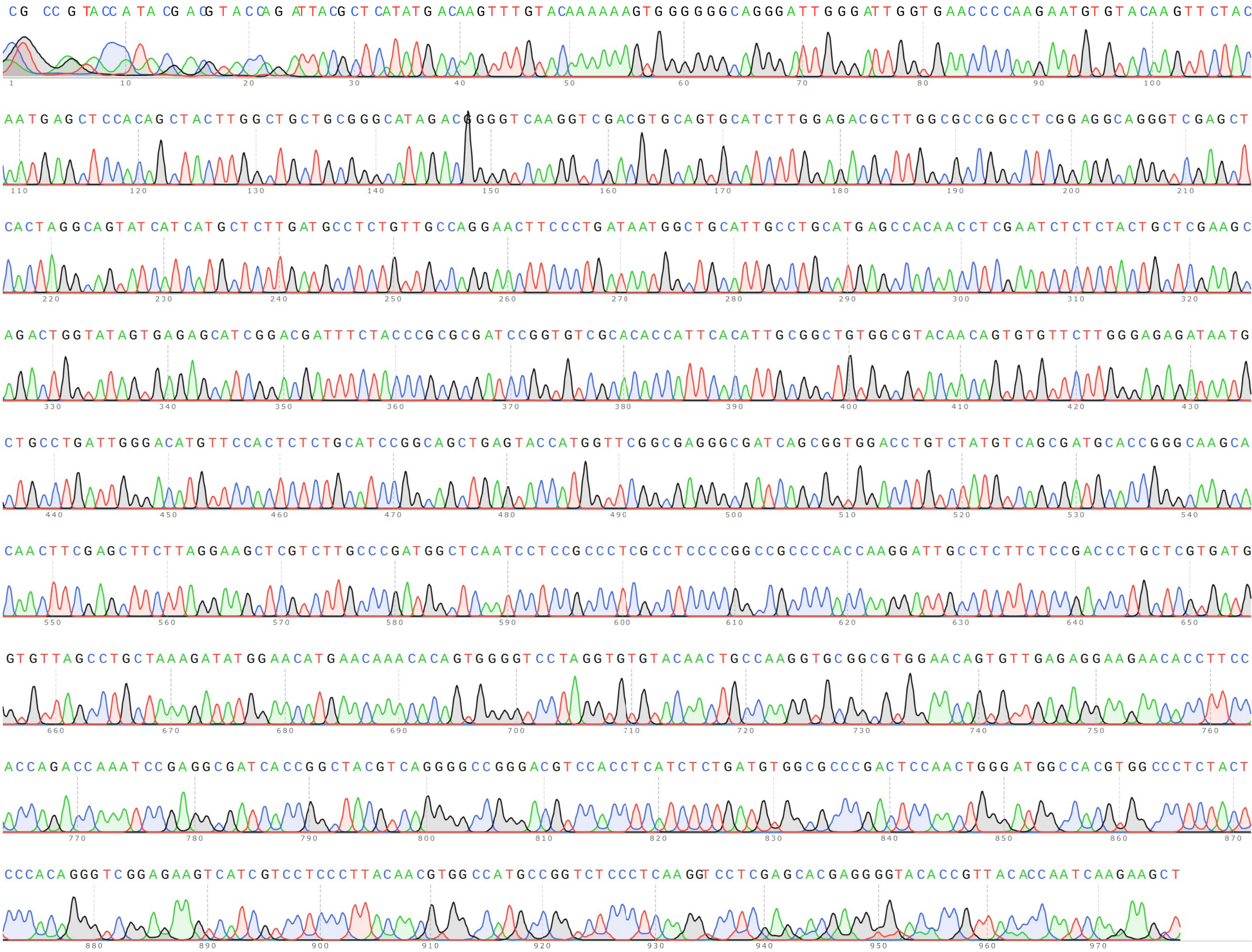
CGCCGTACCATACGACGTACCAGATTACGCTCATATGACAAGTTTGTACAAAAAAGTGGGGGCAGGGATTGGGATTGGTGAACCCCAAGAATGTGTACAAGTTCTAC
1 10 20 30 40 50 60 70 80 90 100
AATGAGCTCCACAGCTACTTGGCTGCTGCGGGCATAGACGGGGTCAAGGTCGACGTGCAGTGCATCTTGGAGACGCTTGGCGCCGGCCTCGGAGGCAGGGTCGAGCT
110 120 130 140 150 160 170 180 190 200 210
CACTAGGCAGTATCATCATGCTCTTGATGCCTCTGTTGCCAGGAACTTCCCTGATAATGGCTGCATTGCCTGCATGAGCCACAACCTCGAATCTCTCTACTGCTCGAAGC
220 230 240 250 260 270 280 290 300 310 320
AGACTGGTATAGTGAGAGCATCGGACGATTTCTACCCGCGCGATCCGGTGTCGCACACCATTCACATTGCGGCTGTGGCGTACAACAGTGTGTTCTTGGGAGAGATAATG
330 340 350 360 370 380 390 400 410 420 430
CTGCCTGATTGGGACATGTTCCACTCTCTGCATCCGGCAGCTGAGTACCATGGTTCGGCGAGGGCGATCAGCGGTGGACCTGTCTATGTCAGCGATGCACCGGGCAAGCA
440 450 460 470 480 490 500 510 520 530 540
CAACTTCGAGCTTCTTAGGAAGCTCGTCTTGCCCGATGGCTCAATCCTCCGCCCTCGCCTCCCCGGCCGCCCCACCAAGGATTGCCTCTTCTCCGACCCTGCTCGTGATG
550 560 570 580 590 600 610 620 630 640 650
GTGTTAGCCTGCTAAAGATATGGAACATGAACAAACACAGTGGGGTCCTAGGTGTGTACAACTGCCAAGGTGCGGCGTGGAACAGTGTTGAGAGGAAGAACACCTTCC
660 670 680 690 700 710 720 730 740 750 760
ACCAGACCAAATCCGAGGCGATCACCGGCTACGTCAGGGGCCGGGACGTCCACCTCATCTCTGATGTGGCGCCCGACTCCAACTGGGATGGCCACGTGGCCCTCTACT
770 780 790 800 810 820 830 840 850 860 870
CCCACAGGGTCGGAGAAGTCATCGTCCTCCCTTACAACGTGGCCATGCCGGTCTCCCTCAAGGTCCTCGAGCACGAGGGGTACACCGTTACACCAATCAAGAAGCT
880 890 900 910 920 930 940 950 960 970
